# Quantitative RNA-seq Analysis Unveils Osmotic and Thermal Adaptation Mechanisms Relevant for Ectoine Production in *Chromohalobacter salexigens*

**DOI:** 10.3389/fmicb.2018.01845

**Published:** 2018-08-13

**Authors:** Manuel Salvador, Montserrat Argandoña, Emilia Naranjo, Francine Piubeli, Joaquín J. Nieto, Lazslo N. Csonka, Carmen Vargas

**Affiliations:** ^1^Department of Microbiology and Parasitology, Faculty of Pharmacy, University of Seville, Seville, Spain; ^2^Faculty of Health and Medical Sciences, University of Surrey, Guildford, United Kingdom; ^3^Department of Biological Sciences, Purdue University, West Lafayette, IN, United States

**Keywords:** ectoines, osmoadaptation, thermoadaptation, RNA-seq, halophile

## Abstract

Quantitative RNA sequencing (RNA-seq) and the complementary phenotypic assays were implemented to investigate the transcriptional responses of *Chromohalobacter salexigens* to osmotic and heat stress. These conditions trigger the synthesis of ectoine and hydroxyectoine, two compatible solutes of biotechnological interest. Our findings revealed that both stresses make a significant impact on *C. salexigens* global physiology. Apart from compatible solute metabolism, the most relevant adaptation mechanisms were related to “oxidative- and protein-folding- stress responses,” “modulation of respiratory chain and related components,” and “ion homeostasis.” A general salt-dependent induction of genes related to the metabolism of ectoines, as well as repression of ectoine degradation genes by temperature, was observed. Different oxidative stress response mechanisms, secondary or primary, were induced at low and high salinity, respectively, and repressed by temperature. A higher sensitivity to H_2_O_2_ was observed at high salinity, regardless of temperature. Low salinity induced genes involved in “protein-folding-stress response,” suggesting disturbance of protein homeostasis. Transcriptional shift of genes encoding three types of respiratory NADH dehydrogenases, ATP synthase, quinone pool, Na^+^/H^+^ antiporters, and sodium-solute symporters, was observed depending on salinity and temperature, suggesting modulation of the components of the respiratory chain and additional systems involved in the generation of H^+^ and/or Na^+^ gradients. Remarkably, the Na^+^ intracellular content remained constant regardless of salinity and temperature. Disturbance of Na^+^- and H^+^-gradients with specific ionophores suggested that both gradients influence ectoine production, but with differences depending on the solute, salinity, and temperature conditions. Flagellum genes were strongly induced by salinity, and further induced by temperature. However, salt-induced cell motility was reduced at high temperature, possibly caused by an alteration of Na^+^ permeability by temperature, as dependence of motility on Na^+^-gradient was observed. The transcriptional induction of genes related to the synthesis and transport of siderophores correlated with a higher siderophore production and intracellular iron content only at low salinity. An excess of iron increased hydroxyectoine accumulation by 20% at high salinity. Conversely, it reduced the intracellular content of ectoines by 50% at high salinity plus high temperature. These findings support the relevance of iron homeostasis for osmoadaptation, thermoadaptation and accumulation of ectoines, in *C. salexigens*.

## Introduction

The halophilic bacteria constitute a group of extremophilic microorganisms that have evolved to survive in hypersaline environments. Despite their NaCl-dependence, these bacteria need osmoadaptation mechanisms to balance the osmotic pressure under different osmotic conditions. Strategies of osmoadaptation were roughly classified into two main types. Extremely halophilic aerobic archaea, bacteria of the order Halanaerobiales, and the extremophile *Salinibacter ruber* use the “salt-in" strategy, which consists of the accumulation of K^+^ and Cl^-^ in the cytoplasm. On the other hand, the “salt-out" or “organic solutes-in" strategy, which involves the intracellular amassment of organic “compatible" solutes, is used by a wider range of microorganisms, including halotolerant and halophilic aerobic bacteria. Compatible solutes are small organic compounds, principally of amino acids and carbohydrate nature, which do not impede cellular metabolism (Brown, 1976; da Costa et al., 1998; Müller et al., 2005).

*Chromohalobacter salexigens* is a halophilic γ-proteobacterium (Arahal et al., 2001) that needs Na^+^ (>0.3 M), Cl^-^ (>0.1 M), and high salinity for optimal growth (Cánovas et al., 1997; O’Connor and Csonka, 2003). In addition, it shows a remarkable salt tolerance (up to 3 M NaCl in a minimal medium; Cánovas et al., 1996), and has been proposed as a model to study prokaryotic osmoadaptation (Ventosa et al., 1998; Vargas et al., 2008). The two main strategies for *C. salexigens* survival under osmotic and heat stress described so far are accumulation of compatible solutes (by synthesis or uptake from the surrounding medium) and membrane adaptation (enhanced synthesis of membrane cardiolipin and cyclopropane fatty acids) (Vargas et al., 2008).

In the absence of external compatible solutes like betaine in the medium, ectoine and hydroxyectoine produced by *C. salexigens* function as the main compatible solutes essential for osmo- and thermo-adaptation, respectively. They are accumulated in response to increasing salinity and temperature (García-Estepa et al., 2006). These solutes are used as protecting agents for macromolecules, cells and tissues, and have a great potential as therapeutic agents for diseases caused by protein misfolding (Pastor et al., 2010).

The halotolerant bacterium *Halomonas elongata* is the well-established microbial host for ectoine production (Lentzen and Schwarz, 2006; Pastor et al., 2010; Kunte et al., 2014). However, *C. salexigens*, either natural or engineered, could be considered as an alternative cell factory (Fallet et al., 2010; Pastor et al., 2010; Rodríguez-Moya et al., 2013; Salvador et al., 2015; Salar-García et al., 2017). In addition to ectoines, *C. salexigens* also synthesizes betaine (from its precursor choline), and minor amounts of glutamate, glutamine, glucosylglycerate, and trehalose (Vargas et al., 2008).

In this work, we used quantitative RNA-seq, complemented with physiological experiments, with two objectives. First, it was for expanding the knowledge about the main transcriptional responses induced by osmotic and heat-stress in this microorganism, others than compatible solutes accumulation. Second, it was for getting insights into how mechanisms involved in osmotic and thermal adaptation could be related to the synthesis of ectoine and hydroxyectoine, in order to optimize the production of these compatible solutes by *C. salexigens* in the future.

## Experimental Procedures

### Bacterial Strain and Growth Conditions

Strain CHR61, a spontaneous rifampicin-resistant mutant of *C. salexigens* DSM 3043, was used as the wild type. *C. salexigens* cells were routinely grown in complex SW-2 medium with 2% (w/v) total salts and 0.5% yeast extract (Nieto et al., 1989). For transcriptomic analysis and phenotypic assays, cells were grown in M63 minimal medium (Csonka, 1982) containing 20 mM glucose as the sole carbon source. Solid media contained an additional 2% Bacto agar (Difco). When used, rifampicin (Rf) was added at a final concentration of 25 μg/ml. pH of SW-2 and M63 media was adjusted to 7.2 with KOH. Cultures were incubated in an orbital shaker at 200 rpm. Growth was monitored as the optical density of the culture at 600 nm (O.D._600_) with a Perkin-Elmer 551S UV/visible spectrophotometer. Because of its halophilic nature, definition of high or low salinity for *C. salexigens* is relative to its optimal salt concentration for growth. As optimal growth occurs between 0.75 and 1.5 M NaCl (Vargas et al., 2006), 0.6 M NaCl is considered as “low salinity,” whereas 2.5 M NaCl is considered as “high salinity.” Both low and high salinity conditions are suboptimal for growth. For all assays, three different growth conditions were used: low salinity (0.6 M NaCl, 37°C), high salinity (2.5 M NaCl, 37°C), and high salinity plus high temperature (2.5 M NaCl, 45°C). Three replicate cultures were grown for each condition. When needed, specific ionophores for Na^+^ (ET2120) and H^+^ (3,3^′^,4^′^,5-tetrachlorosalicylanilide, TCS) were added to the M63 medium at a final concentration of 30 and 0.1 μM, respectively.

### Spectrophotometric Quantification of Glucose

Extracellular glucose was quantified by the Glucose (HK) assay kit (Sigma-Aldrich, St., MO, United States), according to the manufacturer’s recommendations. Measurements were performed in a 96-well microplate reader Synergy HT (BioTek, Winooski, VT, United States).

### RNA Isolation and Ribosomal RNA Removal

Total RNA was isolated from cells using the High Pure RNA isolation kit (Roche). The absence of DNA contamination was checked by PCR using the 16S rRNA primers *16S-RT- fw* and *16S-RT-rv* (Argandoña et al., 2010). After isolation, purity and concentration were assessed in a NanoDrop ND-1000 spectrophotometer (NanoDrop Technologies). Total RNA quality was evaluated by microfluidic capillary electrophoresis on an Agilent 2100 Bioanalyzer (Agilent Technologies) using the Agilent RNA 6000 Nano Kit. Total RNA of high quality [rRNA ratio (23S/16S) ≈1.6 RNA integrity number (RIN) >9.0, and A_260_/A_280_ ratio >2.0] was stored at -80°C until used. Removal of 23S and 16S rRNA was performed using the MICROBExpress^TM^ Bacterial mRNA Enrichment Kit (Ambion) followed by Ribo-Zero rRNA Removal Kit for Gram-negative bacteria (Epicenter). In this way, 99% of the ribosomal RNA was removed from total RNA, ensuring sufficient mRNA to be sequenced. These two sequential rounds of mRNA enrichment did not affect the quality of the resulting mRNA, as described by He et al. (2010). All kits were used according to the manufacturer’s instructions.

### RNA Sequencing

The SOLiD 4 system (Applied Biosystems, Foster City, CA, United States) was used for RNA sequencing (RNA-seq) in this study. SOLiD Total RNA-seq kit was used to fragment, directionally hybridize, and ligate the mRNA with flanking adapters for further PCR amplification and sequencing reactions. The ligated mRNA was converted to cDNA with reverse transcriptase, and the molecules were size-selected following the manufacturer’s instructions. ePCR and emulsion break analyses were performed according to the manufacturer’s manual for the template bead preparation. The beads containing the amplified polynucleotides were first run on a workflow analysis slide to determine the quality and quantity of beads, which was followed by sequencing runs. Quality control of the obtained 50 + 35 nt reads was performed by using the SETS (Solid Experimental Tracking System) software. BioScope version 1.3 (Applied Biosystems) was used to map the color sequences of each bead to the annotated NC_007963 reference genome obtained from the National Center for Biotechnology Information (NCBI). Low quality mapped reads were evaluated and eliminated by using Picard Tools. Once the high quality reads (Phred number >20) were selected, assembling, identification, and quantification was performed by Bayesian inference by using the cufflinks v2.02 software. To assess the genes differentially expressed under the different stress conditions, the HTSeq software^1^ was used to determine the number of reads per gene. The sequencing data obtained in this study have been deposited in NCBI’s Gene Expression Omnibus (Edgar et al., 2002), and are accessible through GEO Series accession number GSE69612.

### Sequencing Data Processing

The EDASeq software from the Bioconductor package (Risso et al., 2011) was used to perform the different normalization procedures. For the differential expression analysis, a negative binomial distribution was assumed using DESeq (Anders and Huber, 2010). Once normalized, the R software^2^ was used to correlate the biological replicates by number of reads per gene. Differentially expressed genes were considered if there was a fold change value greater than 1.70 or lower than -1.70 and a *p*-value adjusted by FDR (“False Discovery Rate”) lower than 0.05 (Benjamini and Hochberg, 1995).

### Semi-Quantitative Reverse Transcription PCR (Semi-qRT-PCR)

SOLiD RNA-seq results were validated using Real Time-PCR as described by Argandoña et al. (2010). Total RNA was used as a template to prepare cDNA by reverse transcription and stored at -20°C until further use. Primer pairs were designed following the methodology described by Argandoña et al. (2010), and are listed in **Supplementary Table S1**. Real-time PCR was performed in 96-well plates using a LightCycler^®^ 480 Real-Time PCR System (Roche) and a FastStart SYBR Green Master (Rox) (Roche). Amplification data were analyzed with the LightCycler^®^ 480 Gene Scanning Software v1.5 (Roche). Transcript levels were calculated by the 2^-ΔΔCT^ method using the mRNA levels of 16S rRNA gene as an endogenous control to normalize the data resulting from each sample.

### Orthology and Ontology Analysis

The Clusters of Orthologous Groups (COGs) functional categories of proteins (Tatusov et al., 1997) were assigned by BLAST against the COG database^3^. Differentially expressed genes were classified automatically according to the KEGG orthology classification (Kanehisa, 1996). To complement this gene classification, differentially expressed genes with annotations from UniProtKB, JCVI (Peterson et al., 2001), NCBI, PATRIC (Wattam et al., 2014), and Ecogene (Zhou and Rudd, 2013) databases were included. Further characterization by ontology was performed by using the Database for Annotation Visualization and Integrated Discovery (DAVID, version 6.7^4^). DAVID calculates a modified Fishers Exact *p*-value to demonstrate GO enrichment, where *p*-values less than 0.05 after Benjamini multiple test correction are considered to be strongly enriched in the annotation category (Huang et al., 2009). This ontology enrichment analysis was used to make a preliminary analysis of the main biological functions of each overexpressed gene set. Potentially interesting clusters identified by DAVID were then manually examined.

### Network Clustering

To complement ontology enrichment analysis, an interaction network was constructed by using the STRING database and the Cytoscape package, followed by ontology network analysis with the BiNGO software. For this purpose, an interaction network was first inferred from the STRING database (Franceschini et al., 2013) by selecting connections over a threshold of 0.7 of confidence value. This network was condensed and clustered into the most relevant groups (5% over- or under-represented) that were differentially overexpressed at each condition, by using the ExprEssence software (Warsow et al., 2010) of the Cytoscape platform (Shannon et al., 2003). Afterward, the derived networks were submitted to further ontology analysis by BiNGO (Maere et al., 2005) by using the *C. salexigens* UniProtKB gene ontology. The resulting networks represent the set of functional clusters overexpressed at each experimental condition.

### Analytical Determinations of Intracellular Content of Iron and Sodium

The intracellular concentrations of iron and sodium were determined as described previously by Becerra et al. (2014) and Robert et al. (2000), respectively. To determinate the iron content, the *C. salexigens* wild type strain was grown until mid-logarithmic phase in M63 medium supplemented with 20 mM glucose at different salinities (0.6 or 2.5 M Ultrapure NaCl (Sigma) and temperatures (37 or 45°C), in the presence or absence of 50 μM FeCl_3_. Then, cells from 5 ml of culture were harvested by centrifugation at 12,000 *g* for 5 min, and the cell pellet was washed three times with the same volume of iron-free M63 medium (Argandoña et al., 2010). Subsequently, cell pellets were dried at 70°C until weight became constant to ensure total removal of water, and the dry cell weight (DCW) was estimated. Finally, the iron content was determined using an Ultima 2 inductively coupled plasma optical emission spectrometer (ICP-OES; HORIBA Jobin Yvon). For the quantification of the intracellular sodium content, a similar procedure was carried out but using Na^+^-free M63 medium where NaCl was replaced with KCl to wash the cell pellets.

### Siderophore Production Assay

To determinate siderophore production by *C. salexigens*, the procedure described by Schwyn and Neilands (1987) and modified by Argandoña et al. (2010), was followed to prepare Chrome Azurol S (CAS) agar plates. The *C. salexigens* wild type strain was grown at 37°C in M63 medium containing 20 mM glucose at the three conditions tested for RNA-seq experiments, until mid-logarithmic phase. One milliliter of each culture was centrifuged, washed with the same medium to remove siderophores, and resuspended in 30 μl of M63 medium containing 0.6 or 2.5 M NaCl. Aliquots of 10 μl were placed on the corresponding modified CAS agar plates supplemented with different concentrations of NaCl, and incubated for 72 h at 37 or 45°C. CAS is both a low-iron affinity chelating agent and an indicator. It is blue-green when it is chelated with iron and turns orange when Fe is removed from it by higher-affinity chelating agents present in the medium, like siderophores. The diameter of the halo is proportional to the quantity of siderophores secreted by the cell.

### LC-MS Quantification of Ectoine and Hydroxyectoine

One milliliter of cell culture at late exponential phase was used for liquid chromatography-mass spectrometry (LC-MS) analysis of intracellular content of ectoine and hydroxyectoine. The compatible solutes were extracted by using a modified Bligh and Dyer (1959) technique as described by Kraegeloh and Kunte (2002). Chromatographic separation and MS quantification of ectoine and hydroxyectoine was performed as described by Argandoña et al. (2010). Concentration of each compatible solute was expressed as μmol solute/g DCW.

### H_2_O_2_ Sensitivity Assays

Sensitivity to hydrogen peroxide (H_2_O_2_) was determined at the three conditions tested for RNA-seq experiments. Cells were grown in tubes with SW-2 medium at 37°C and 200 rpm until O.D._600_ = 1.6, and then transferred to tubes with 5 ml of 20 mM glucose M63 medium with the corresponding NaCl concentration (0.6 or 2.5 M), and incubated at 37 or 45°C and 200 rpm until mid-logarithmic phase. Then, 50 μl of each culture was inoculated to 5 ml of M63 medium containing 20 mM glucose, 0.6 or 2.5 M NaCl, and H_2_O_2_ at increasing concentrations (0–30 μM) adjusted by 3% H_2_O_2_ water. Subsequently, cultures were incubated for 24 or 48 h at 37 or 45°C and 200 rpm, and the minimal concentration of H_2_O_2_ able to inhibit growth was determined.

### Swarming Motility Assay in Semisolid Agar Plates

Swarming motility assays were based on the procedure previously described by Wolfe and Berg (1989). Cells were grown in tubes with SW-2 medium at 37°C and 200 rpm until O.D._600_ = 1.6. Then, 50 μl of cultures were transferred to tubes with 5 ml of M63 medium containing 20 mM glucose and 0.6 or 2.5 M NaCl, and incubated at 37 or 45°C and 200 rpm until mid-logarithmic phase. Swarming plates of 20 mM glucose M63 medium (with 0.6 or 2.5 M NaCl) were prepared with 0.35% agar. A 3-μl aliquot of the appropriate culture was placed on the center of the plates by clicking the agar with the tip. The plates were incubated at 37 or 45°C in a humid environment. The migration halo was monitored during 163 h and the swarming radii (mm) were measured. Duplicated plates were prepared to avoid artifacts due to changes in temperature that might occur by handling. If needed, specific ionophores EHT2120 or TCS were added at a final concentration of 30 and 0.1 μM, respectively.

## Results and Discussion

### Sequencing of *C. salexigens* Transcriptome

To investigate the transcriptional response of *C. salexigens* to osmotic and heat stress, in conditions for optimal and suboptimal ectoine production, nine RNA-seq libraries were generated from *C. salexigens* grown in three different conditions: low salinity (0.6 M NaCl at 37°C; low production of ectoines), high salinity (2.5 M NaCl at 37°C; high production of ectoine), and high salinity plus high temperature (2.5 M NaCl at 45°C; high production of hydroxyectoine) (see Experimental Procedures). Cell samples were collected during exponential phase when cultures reached enough biomass (low salinity: 0.8655 ± 0.1619 g/L; high salinity: 1.4208 ± 0.1618 g/L; high salinity and temperature: 1.6017 ± 0.0225 g/L) to assure isolation of 100–500 ng of mRNA for RNA-seq library construction. A 99.7% ± 0.067 removal of rRNA was performed to ensure enrichment of whole transcriptome and good coverage in RNA-seq. In addition, sampling was carried out before glucose was depleted, to avoid cells consuming extracellular by-products accumulated during exponential growth (Pastor et al., 2013; Salvador et al., 2015; **Supplementary Figure S1**). RNA-seq analysis was performed on three biological replicates of *C. salexigens* wild type cells grown in each condition, using the SOLiD^TM^ v4 system (see Experimental Procedures).

An average of ∼77.23 million reads was obtained per sample and low quality mapped reads were evaluated and eliminated. About 74.56% of total reads were mapped to the annotated *C. salexigens* genome, accession number NC_007963.1 (Copeland et al., 2011; see **Supplementary Table S2**) and matched with the majority of *C. salexigens* open reading frames. The only exceptions were *csal3049* (not detected in any of the conditions tested); *csal1393*, *csal1394*, *csal2367*, and *csal2259* (not detected at low salinity); *csal1190* (not detected at high salinity); and *csal0649* and *csal1409* (not detected at high salinity plus high temperature). The predicted products of these genes were annotated as hypothetical proteins except for *csal1409*, assigned to a pseudogene; *csal1394*, annotated as a potential phage protein; and *csal0649*, annotated as a short-chain dehydrogenase.

### Data Normalization and Validation of Differentially Expressed Genes

Due to the nature of the RNA-seq analysis, strong deviations in results might be due to differences in the length of the gene and the size of the library in each sample. Numerous studies stress the importance of standardizing the data for the removal of different statistical deviations that can distort all subsequent analyzes (Risso et al., 2011; Hansen et al., 2012). Normalization was performed using the variance analysis package DESeq (Anders and Huber, 2010), followed by calculation of the *p*-value to determine the statistical significance among the number of reads per gene along the biological samples. When expression data for each replicate were plotted against each other, the different sequencing runs were found to be highly reproducible, with Pearson correlation coefficients greater than 0.9. This confirmed that the biological replicates were adequately reproducible (Tan et al., 2003; **Supplementary Table S3**).

**FIGURE 1:**
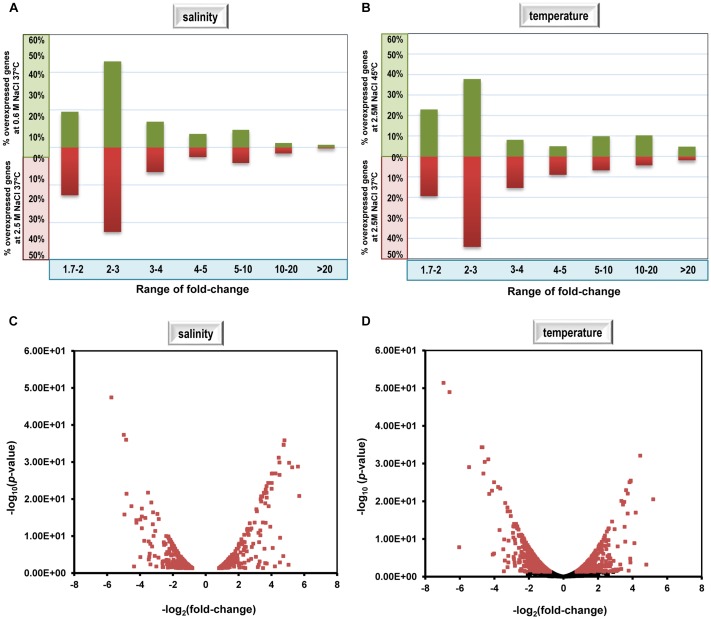
Percentage of genes included in fold-change ranges and volcano plot of differential transcriptome data of *C. salexigens* at 0.6 M NaCl at 37°C versus 2.5 M NaCl at 37°C **(A,C)**, and at 2.5 M NaCl at 37°C versus 2.5 M NaCl at 45°C, **(B,D)**. In volcano plot graphs, points in red represent differentially expressed genes with fold change >1.7 and adjusted *p*-value for “false discovery rate” (FDR) <0.05. Positive values correspond to genes overexpressed at 2.5 M NaCl at 37°C **(C)** and at 2.5 M NaCl at 45°C **(D)**, and negative values correspond to genes overexpressed at 0.6 M NaCl at 37°C **(C)** and at 2.5 M NaCl at 37°C **(D)**.

About 60% of *C. salexigens* differentially expressed genes fell within the range of 2–4 fold-changes (*p*-value < 0.05) (**Figure 1**). Additionally, it was found that most of differentially expressed genes within the 1.7–1.99 fold-change belonged to same operons of those with a higher fold-change, or had the biological functions related to those with a higher fold-change. Therefore, for the purpose of this study, differentially expressed genes with a fold change value greater than 1.7 or lower than -1.7, and a *p*-value lower than 0.05 were considered. Our RNA-seq analysis identified a total of 1,081 genes (31.88% of *C. salexigens* genome) whose transcript levels responded to increases or decreases in the external salinity. 539 of those genes were induced at low salinity and 542 were induced at high salinity. Analysis of the differential expression patterns for cells grown at 37°C versus 45°C in 2.5 M NaCl revealed a total of 662 genes (19.52% of total the genome), of which 287 were induced and 375 were repressed by high temperature (**Figure 1**).

To validate the RNA-seq results, we selected genes related to metabolism and uptake of ectoine (*doeA*, *doeD*, *eutC*, *teaA*, and *teaC*) and cyclopropane fatty acid synthesis (*cfa1* and *cfa2*), which were found to be differentially expressed by RNA-seq, and measured their expression under the same experimental conditions by semi-qRT-PCR. A high Pearson correlation (0.85) was found between the two methods (**Supplementary Figure S2**). In addition, the qualitative pattern of regulation of these genes by RNA-seq was identical to those measured by semi-qRT-PCR, leading to the validation of the RNA-seq results for comparative transcriptome analysis.

### Global Transcriptional Response to Osmotic and Heat Stress

**FIGURE 2:**
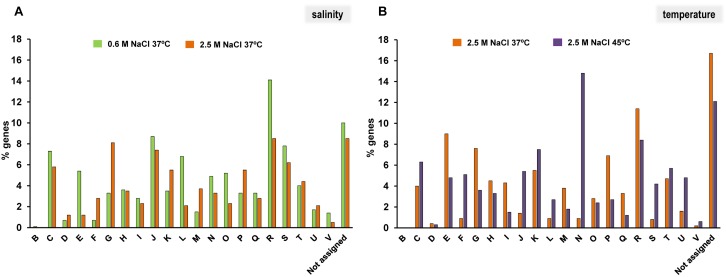
Percentages of functional categories assigned by COG of differentially expressed genes in *C. salexigens*. **(A)** Percentages of genes induced at 0.6 M/2.5 M NaCl (green) and 2.5 M NaCl/0.6 M NaCl (orange) at 37°C. **(B)** Percentages of genes induced at 37°C/45°C (orange) and at 45°C/37°C (purple) at 2.5 M NaCl. The COG categories are as follows: B, chromatin structure and dynamics; C, energy production and conversion; D, cell cycle control, cell division, chromosome partitioning; E, amino acid transport and metabolism; F, nucleotide transport and metabolism; G, carbohydrate transport and metabolism; H, coenzyme transport and metabolism; I, lipid transport and metabolism; J, translation, ribosomal structure and biogenesis; K, transcription; L, replication recombination and repair; M, cell wall/membrane/envelope biogenesis; N, cell motility; O, post-translational modification, protein turnover, chaperones; P, inorganic ion transport and metabolism; Q, secondary metabolites biosynthesis, transport and catabolism; R, general function prediction only; S, function unknown; T, signal transduction mechanisms; U, intracellular trafficking, secretion and vesicular transport; V, defense mechanisms; and Not assigned, not in COGs.

Due to the large number of differentially transcribed genes, we first analyzed the functional categories of their encoded products using the COG database. Genes encoding proteins that belonged to 21 functional categories were overexpressed, including those that are related to general bioprocesses or specific functions (**Figure 2**). It is important to highlight that there was a large number of genes that did not belong to a specific COG, or were not assigned to a particular one (**Figures 2A,B**). Interestingly, some functional categories increased their representation at each experimental condition. Thus, at low salinity, functional categories related to amino acid transport and metabolism (E), replication, recombination and repair (L), post-transcriptional modification and turnover and chaperonins (O), or defense mechanism (V) were the most highly represented (**Figure 2A**). In contrast, functional categories involved in transport and metabolism of nucleotides (F) and carbohydrates (G), transcription (K), cell wall, membrane and envelope biogenesis (M) and inorganic ion transport and metabolism (P) were the most abundant categories of genes overexpressed at high salinity at 37°C compared to 0.6 M at 37°C (**Figure 2A**). However, when 2.5 M at 37°C was compared to high salinity at 45°C, the most highly represented categories were amino acid (E), carbohydrate (G) and lipid (I) transport and metabolism, cell wall, membrane and envelope biogenesis (M), and inorganic ion transport and metabolism (P) (**Figure 2B**). In contrast, genes assigned to categories involved in energy production and conversion (C), translation, ribosomal structure and biogenesis (J), replication, recombination and repair (L), cell motility (N), and intracellular trafficking, secretion and vesicular transport (U) further increased their representation in response to temperature increase (**Figure 2B**). These findings suggest that both osmotic and heat stresses have a significant influence on *C. salexigens* transcriptome, and consequently on its global physiology.

To better understand *C. salexigens* global transcriptional responses to osmotic and heat stress, differentially expressed genes were submitted to refinement and functional clustering analysis (see Experimental Procedures). This allowed us to identify the main functional gene clusters that might be involved in *C. salexigens* osmo- and heat-stress adaptive responses (**Supplementary Tables S4, S5** and **Supplementary Figure S3**). The most relevant results are discussed below.

### Transcriptional Response Related to the Metabolism of Ectoines and Other Compatible Solutes

For adaptation to high salinity or high temperature environments, bacteria depend on *de novo* synthesis or uptake of compatible solutes (Roberts, 2006). The TRAP-like TeaABC osmoregulated transporter involved in the specific uptake of ectoine and hydroxyectoine was first described in *H. elongata.* This system is also implicated in keeping a constant intracellular ectoine content in the absence of external solutes, by rescuing ectoine escaped through the cytoplasmic membrane (Grammann et al., 2002). Additionally, in *H. elongata* the two first enzymes involved in the ectoine degradation route (DoeA and DoeB), together with the ectoine synthesis enzymes EctA and EctC, are suggested to constitute a recycling pathway that functions to finely adjust the internal concentration of ectoines in response to external changes in osmolarity (Schwibbert et al., 2011).

Although RNA-seq samples were taken in “*de novo* synthesis conditions” (minimal medium with glucose as sole carbon source), high salinity induced genes encoding ATP-binding-cassette (ABC) transport systems for betaine and choline, and the tripartite ATP-independent transport system (TRAP) for ectoines (*teaABC*) (Grammann et al., 2002) (**Supplementary Table S6**). At high salinity, overexpression of up to seven genes orthologous to those encoding putative or characterized choline-O-sulfate and/or glycine-betaine- (CbcX and OpuAC; Kempf et al., 1997; Nau-Wagner et al., 1999; Chen et al., 2010; Malek et al., 2011) or ectoine-binding proteins (TeaA; Grammann et al., 2002) was observed. Therefore, although extracellular betaine or its precursor choline suppresses the synthesis of ectoines in *C. salexigens* (Calderón et al., 2004; Vargas et al., 2006), the induction of *de novo* synthesis of ectoines at high salinity (i.e., in cells grown in minimal medium) did not inhibit expression of genes encoding uptake systems for other compatible solutes. These findings are in agreement with the common presumption that, under osmotic stress, uptake of osmoprotectants is favored over the synthesis, because it is energetically cheaper to the cells (Oren, 1999).

High temperature repressed the expression of genes orthologous to characterized binding proteins involved in the uptake of glycine-betaine and/or choline-sulfate (CbcX, OpuAC, OsmX, BetT, and YehZ; Kempf et al., 1997; Andresen et al., 1998; Nau-Wagner et al., 1999; Chen et al., 2010; Malek et al., 2011; Frossard et al., 2012; Lang et al., 2015), as well as the *teaABC*. In contrast, the gene *betI*, encoding the predicted repressor of betaine transport (Lamark et al., 1996), was temperature-induced (**Supplementary Table S6**). These results were somehow surprising, as heat-induced hydroxyectoine synthesis confers thermo-protection to *C. salexigens* (García-Estepa et al., 2006) and external betaine and choline sulfate function as thermoprotectants for *E. coli* (Caldas et al., 1999). On the other hand, exogenously added ectoine and hydroxyectoine confer both osmo- and thermoprotection to *Streptomyces coelicolor*. In this microorganism, uptake of radiolabeled ectoine was triggered by either salt or heat stress, and the highest level of uptake was found in cells subjected to both stresses (Bursy et al., 2008). One hypothesis is that in *C. salexigens* transport of compatible solutes might be active at high temperature, despite transcriptional inhibition. However, there is not experimental evidence for this. Alternatively, transcriptional inhibition, and concomitant lower activity, of transport systems by temperature, may suggest that under heat stress the uptake of compatible solutes is not as advantageous as their synthesis (i.e., ATP is needed), or that the uptake process is lees effective in these conditions (i.e., because of alteration of Na^+^- or H^+^-gradients by temperature). In this regard, the Cbc, Opu, Osm, and Yeh uptake systems are ABC-type and recruit ATP for compatible solutes transport. Furthermore, TeaABC (TRAP-type), and BetT (BCCT-type) uptake systems are Na^+^- and H^+^-dependent, respectively, so they are energized by SMF (Sodium Motive Force) or PMF (Proton Motive Force).

Regarding compatible solutes synthesis, two betaine aldehyde dehydrogenase genes (*csal1515* and *csal1706)*, as well as genes for the synthesis of betaine from choline (*csal_2844)*, the ectoine’s precursor aspartate semialdehyde (*ask)*, and the synthesis of hydroxyectoine from ectoine (*ectD*), were induced at high salinity (**Supplementary Figure S3B** and **Supplementary Table S6**). Up-regulation of *ectD* with salinity correlated with our previous results of hydroxyectoine accumulation (García-Estepa et al., 2006). However, induction of the ectoine synthesis gene *ectA*, previously observed by qPCR (Argandoña et al., 2010), was not detected in this work. In conditions of osmotic and thermal stress (high production of hydroxyectoine), the *ask* gene encoding aspartate semialdehyde synthesis was down-regulated. Interestingly, the ectoine hydroxylase gene *ectD* was not further induced by high temperature (**Supplementary Table S6**), suggesting that the induction of hydroxyectoine synthesis observed in *C. salexigens* in response to heat stress (García-Estepa et al., 2006) could be controlled mainly at the post-transcriptional level.

Concerning catabolism of ectoines, genes orthologous to *doeABCD* (involved in degradation or recycling of ectoine in *H. elongata*; Schwibbert et al., 2011) and *eutBC* (involved in hydroxyectoine degradation in *Ruegeria pomeroyi* DSS-3; Schulz et al., 2017) were induced at high salinity (**Supplementary Table S6**). Genes *doeB*, *doeC*, and *doeD* were down-regulated by high temperature (**Supplementary Table S6**). In contrast, in *H. elongata* *doeA*, *doeB*, and *doeD* were repressed by salinity (Kindzierski et al., 2017), suggesting metabolic differences between both microorganisms, at least in salinity adaptation. *C. salexigens* uses ectoines as the sole carbon sources only at optimal and high salinity (Rodríguez-Moya et al., 2013). Although the RNA-seq data were related to cells grown on glucose, they suggest that glucose does not repress expression of the ectoine catabolic genes at high salinity. In fact, ectoine catabolism, measured as the fate of [^14^C]-ectoine, is only partially repressed in the presence of glucose (Vargas et al., 2006).

On the other hand, *C. salexigens* genes *doeB*, *doeC*, and *doeD* were down-regulated by high temperature (**Supplementary Table S6**), suggesting a dual transcriptional control by salinity and temperature of these genes. No experimental data are available about the influence of temperature in ectoine catabolism in *C. salexigens*. Therefore, despite the transcriptional repression, we cannot infer that catabolism of ectoines in *C. salexigens* is inactive at high temperature.

Altogether, the above results suggest that ectoine metabolic routes (synthesis, uptake and catabolism) might work co-ordinately to ensure the appropriate solute pool with optimal cellular resources. In fact, genes for the uptake and catabolism (or recycling) of ectoines seem to be co-regulated by salinity and temperature. Our findings also suggest a different regulatory mechanism, or perhaps a different role, for the ectoine degradation/recycling pathway in *H. elongata* and *C. salexigens*. Interestingly, deletion of *teaC* and *doeA* in *H. elongata* led mutants to overproduce ectoine at high salinity (Schwibbert et al., 2011). Thus, the influence of osmotic and heat stress on the transcriptional control of the pathways involved in metabolism of ectoines, as well as the existence of post-transcriptional control mechanisms, deserve further investigation. This knowledge could be applied to design new engineering strategies to generate ectoine-overproducing strains.

### Salt- and Heat-Dependent Variations of the Transcriptional Oxidative Stress Response

Network clustering and DAVID ontology enrichment analyses, followed by an additional exhaustive search for genes related to the oxidative stress response, revealed differential expression of genes encoding primary or secondary oxidative stress mechanisms, depending on the experimental conditions tested. Induction of secondary repair mechanisms was mainly observed at low salinity (0.6 M NaCl, 37°C), whereas a primary oxidative stress response was mostly induced at high salinity (2.5 M NaCl, 37°C) and repressed by temperature (2.5 M NaCl, 45°C).

At low salinity, genes coding for protein repair systems like a thioredoxin reductase (*csal2959*), a DsbA-like periplasmic oxidoreductase (*csal3118*), a glutaredoxin (*csal1255*), and a methionine sulfoxide reductases (*csal1350* and *csal2656*), were induced (**Supplementary Figure S3** and **Supplementary Table S7**). In addition, genes encoding redox balance-maintaining mechanisms, such as those related to glutathione metabolism (i.e., the dioxygenases *csal0130*, *csal0135*) and related to direct regeneration of reducing equivalents (i.e., the FAD-dependent pyridine nucleotide-disulfide oxidoreductase *csal0136*), were up-regulated. Genes participating in DNA repair including exo- and endonucleases, the Rec homologous recombination system, and error-prone DNA polymerases (i.e., *csal1275*) were also overexpressed (**Supplementary Table S7**). Interestingly, genes for cytochrome bd terminal oxidase (*csal2000*-*csal2001*) were induced in this condition (see **Supplementary Table S9**). Beyond its role in cell bioenergetics, in phylogenetically distant bacteria such as *Escherichia coli* (Giuffrè et al., 2014) and the Gram-positive, thermophilic, acetogenic *Moorella thermoacetica* (Das et al., 2005), this terminal oxidase is involved in the tolerance to oxidative stress, owing to its remarkable catalase activity.

High salinity induced the expression of up to five genes encoding direct ROS (Reactive Oxygen Species) detoxifying enzymatic mechanisms: a catalase orthologous to KatG (*csal0159*), an iron superoxide dismutase (*csal1861*), the peroxidase OsmC (*csal0037)*, a 1-Cys peroxiredoxin (*csal0179*), and an alky-hydroperoxide reductase (*csal0321*) (**Supplementary Table S7** and **Supplementary Figure S3**). A different set of genes encoding redox balance-maintaining mechanisms related to glutathione metabolism (i.e., the dioxygenases *csal2376* and *csal3007*) were also induced. In addition, a number of genes with secondary functions in oxidative damage repair were overexpressed. These include genes encoding protein repair systems, such as glutaredoxins (*csal0377* and *csal2129*) and the iron-sulfur cluster regulator IscR (*csal2847*) (**Supplementary Table S7**).

Interestingly, high temperature attenuated the salt-induced transcriptional primary oxidative stress response (**Supplementary Table S7** and **Supplementary Figure S3**). These included genes encoding a KatE-like catalase (*csal0803*), an alkyl-hydroperoxide reductase (*csal1130*), the copper/zinc binding superoxide dismutase *csal0397*), and the osmotically induced genes for the peroxidases OsmC (*csal0037)* and peroxiredoxin (*csal0179*). In addition, genes encoding a redox balance dioxygenase (*csal2376*), a glutaredoxin (*csal2129*), and the DNA repair protein RadC (*csal2972*), were down-regulated. In contrast, genes encoding detoxification systems for reactive oxygen species related to nitrogen-containing molecules, as NorR (*csal0451*), were induced (**Supplementary Table S7**).

The above results led us to investigate a possible salt-induced cross-protection mechanism against oxidative stress. For this purpose, we inspected *C. salexigens* resistance to oxidative stress (imposed by increasing concentrations of H_2_O_2_) in the same conditions of RNA-seq. As shown in **Table 1**, *C. salexigens* was much more sensitive to oxidative stress at high salinity than at low salinity, as no growth was observed above 5 μM H_2_O_2_ at 2.5 M NaCl, regardless of temperature. This finding suggests that osmotic stress does not confer protection against damage by oxidative stress in *C. salexigens*, in spite of the salt-induced strong transcriptional primary oxidative stress response. This was not the case of the non-halophilic bacterium, food-borne pathogen, *Campylobacter jejuni*, where upregulation of oxidative stress genes under hyperosmotic stress resulted in a moderate protection against oxidative stress (Cameron et al., 2012).

**Table 1:** Influence of salinity and temperature on *C. salexigens* resistance to H_2_O_2_.

H_2_O_2_NaCl/T^a^	0 μM	5 μM	10 μM	15 μM	20 μM	25 μM	30 μM
0.6 M 37^o^C	+	+	+	+	+	+	-
2.5 M 37^o^C	+	-	-	-	-	-	-
2.5 M 45^o^C	+	-	-	-	-	-	-

*+, Growth; -, No growth.*

The induction of different oxidative stress response mechanisms, and the distinct sensitivity to H_2_O_2_, might point out different factors causing oxidative stress at low and high salinity in *C. salexigens*. The induction of genes encoding iron-chelating agents (**Supplementary Table S11**), and DNA and protein repair systems at low salinity suggested that a higher intracellular iron concentration, probably due to a higher demand for iron in this condition (Argandoña et al., 2010) might cause (at least part of) the oxidative stress faced by *C. salexigens* at low salinity. This correlation of the secondary oxidative stress response with iron homeostasis will be further investigated and discussed below.

The salt-induced oxidative stress at high salinity might be caused by a direct effect of salt-induced ROS generation, and/or be related to differences in respiration rate or cell redox state. The *in silico* metabolic flux analysis of *C. salexigens* osmoadaptation recently reported by our group (Piubeli et al., 2018) suggests that the improvement in metabolic efficiency experimentally observed at high salinity (Pastor et al., 2013) could be driven by an increment in the respiration rate. These *in silico* and experimental analyses also suggested that both the Entner-Doudoroff pathway and the TCA cycle are more active at high salinity, in order to support ATP production, carbon (TCA intermediaries) and reducing equivalents, which are necessary for ectoine production (Pastor et al., 2013; Piubeli et al., 2018). In addition, the two alternative pathways for glucose metabolism (phosphorylative or non-phosphorylative branches of Entner-Doudoroff pathway) seemed to be differently used by *C. salexigens* depending on salinity, in order to control the production rate of redox equivalents, and finely tune the cell redox state to maximize growth and biosynthesis of ectoines (Pastor et al., 2013). These two findings could be related to an increment of oxidative stress at high salinity.

Finally, the attenuation of the salt-induced transcriptional oxidative stress response at high temperature was very intriguing. In the halophilic non-ectoine producing bacterium *Jeotgalibacillus malaysiensis* (Yaakop et al., 2016), a rise in NaCl concentration (that is expected to generate high ROS concentration) led to a down-regulation of catalase and peroxidase. The authors suggested that to counteract perturbations in the cytoplasmic redox state, this organism re-routes carbohydrate flux from the glycolysis to the pentose phosphate pathway by increasing cellular levels of an antioxidant compound, i.e., the cofactor NADPH. Hydroxyectoine has an *in vitro* moderate antioxidant capacity (Andersson et al., 2000) and becomes predominant at stationary phase (where oxidative stress levels are more pronounced; Fallet et al., 2010), and in response to heat stress (García-Estepa et al., 2006). The hypothesis that at high salinity and temperature the accumulation of hydroxyectoine could protect *C. salexigens* against the salt-induced oxidative stress (as the NAPDH for *J. malaysiensis*) with attenuating the oxidative stress response, needs experimental confirmation.

### Transcriptional Response of Protein-Folding Stress Related Genes

Physical and chemical factors such as heat, salinity, oxidative stress, solvents, heavy metals, or antibiotics, can disrupt protein homeostasis and lead to the amassing of misfolded proteins, which may end in an aggregated stage (Mogk et al., 2011). For proteostasis, bacteria have developed sophisticated responses to folding stress involving chaperones and proteases (Mogk et al., 2011; Lee et al., 2016). Interestingly, the gene *csal2986*, encoding the key regulator of the heat stress response RpoH (Tilly et al., 1986) was induced at low salinity at 37°C. In addition, a large number of genes for heat shock proteins, various chaperones, and proteases, some of which belong to the RpoH regulon, were induced (**Supplementary Table S8** and **Supplementary Figure S3A**). These included genes for the DnaK/DnaJ chaperone system (*csal3093*/*csal3094*), involved in assisting folding of newly synthesized proteins and preventing the aggregation of misfolded proteins under environmental stress, as well as genes for enzymes belonging to the AAA+ family of proteases. The latter ones have been reported to play a vital role in the removal of misfolded proteins in favor of cell homeostasis (Mogk et al., 2011). Surprisingly, at high salinity and temperature only the genes for heat shock protein Hsp90 (*csal2497*) and a DnaJ-like protein (*csal0100*) were induced, whereas those encoding the protease ClpA (*csal2440*) and a different chaperone of the DnaJ family (*csal1428*) were repressed (**Supplementary Table S8**).

The above findings strongly suggest that at low salinity *C. salexigens* is facing disruption of protein homeostasis. The physiological basis for this is unknown, but the phenomenon does not seem to be exclusive to this organism, as it was also observed in the halotolerant *Vibrio parahaemolyticus* and *V. vulnificus* (Yang et al., 2010; Rao et al., 2013). It has been suggested that cytoplasmic proteins of *C. salexigens* and other compatible solute-accumulating halophilic bacteria evolved to function optimally in the presence of high concentrations of osmolytes. Thus, *C. salexigens* cytoplasmic proteins are “slightly halophilic,” as they expose at the surfaces more acidic amino acids than those of non-halophilic bacteria (Oren et al., 2005). As ectoines are zwitterionic, this surface remodeling would contribute to maintaining the solubility and stability of proteins in solutions containing high concentrations of ectoines (Siglioccolo et al., 2011). Thus, it is possible that at low salinity proteins would tend to misfold because of the inadequate concentration of compatible solutes, eliciting protein-folding stress. On the contrary, at high salinity (even in presence of a strong oxidative stress), massive accumulation of ectoines would assume the chaperone role of the heat-shock proteins, and promote protein folding, solubility, and stability.

Contrary to *C. salexigens*, in the halophilic bacterium *J. malaysiensis* high salinity induced genes encoding molecular chaperons (ClpB, a co-chaperon of DnaK), heat/shock proteins or proteases (DegA), as well as genes for enzymes involved in repairing oxidized proteins. Remarkably, this bacterium preferentially accumulates proline, glutamate, or trehalose in response to salinity, but not ectoines (Yaakop et al., 2016). These discrepancies suggest that transcriptional response to protein folding stress, which was found to be induced by osmotic stress in *C. salexigens*, could be systematically different from that in other halophilic microorganisms.

### Transcriptional Response of Respiratory Chain and Electrochemical Gradients to Osmotic and Heat Stress

*Chromohalobacter salexigens* is a strictly aerobic and cytochrome c oxidase-negative bacterium (Arahal et al., 2001). Accordingly, there are no orthologues of genes encoding cytochrome caa_3_ terminal oxidases in its genome. Instead, genes for the cytochromes bd (*csal2000*-*csal2001*) and bo (*csal0954*-*csal0957*) ubiquinol terminal oxidases are present, suggesting that *C. salexigens* has a branched respiratory chain. Remarkably, the genome of *C. salexigens* contains genes that potentially encode the three NAD(P)H dehydrogenases known in prokaryotes (see below). Transcriptional responses observed by RNA-seq suggested that salinity and temperature induced shifts in the respiratory chain and associated components (**Supplementary Table S9**).

Differentially overexpressed genes at low salinity included those encoding the terminal oxidase cytochrome bd (*csal2000* and *csal2001*) and its regulator RegA (*csal1826*), the electron-transferring-flavoprotein dehydrogenase units (*csal1612* and *csal1614*), and the cytochrome b561-like enzymes (*csal1313* and *csal1512*), which may be coupled to the cytochrome bd or cytochrome bo terminal oxidases, as well as the non-pumping NAD(P)H dehydrogenase (quinone) (*csal2801*, homologous to NDH-II). These transcriptional changes were associated with induction of genes encoding quinone-related enzymes or components, such as ubiquinone and quinone synthesis enzymes (*csal0595*, *csal0912*, *csal0845*, *csal0848*, and *csal2863*), a malate:quinone oxidoreductase (Mqo and *csal2579*) and a gluconolactonase (Glnl) (*csal2779*) (**Supplementary Table S9**). Furthermore, the gene encoding RegA (*csal1826*), a response regulator of a two-component system that senses the balance among oxidized quinones (Wu and Bauer, 2010), was induced at low salinity. These results agree with our previous finding that *C. salexigens* has a certain preference for using enzymes which reduce quinones over NAD at low salinity. This mechanism was proposed as an alternative redox balance system used when intracellular concentration of ectoines is low (Pastor et al., 2013; Salvador et al., 2015).

High salinity induced the expression of genes encoding the proton-pumping- NADH dehydrogenase complex I (*csal3121* to *csal3132*, similar to NDH-I) (NAD-I) and a F0F1 ATP synthase (*csal3283-3289*) (**Supplementary Table S9**). Using a high-quality genome based metabolic model and Flux Balance Analysis (FBA), we have recently showed that the *C. salexigens* ATP synthase complex subunits genes were only essential at high salinity. Additionally, the theoretical ATP yield at high salinity was higher compared to low salinity, and mainly produced through oxidative phosphorylation (Piubeli et al., 2018). The finding that the ATP synthase subunits genes were overexpressed at high salinity, gives support to the relevant role of bioenergetics in the metabolic adaptations to salinity in this microorganism. This would correlate with the higher biomass and ectoine production observed experimentally at high salinity (Pastor et al., 2013).

Interestingly, at high salinity plus high temperature genes encoding components of the respiration-driven primary sodium-pumping NADH:ubiquinone oxidoreductase (Na^+^-NQR; *csal1569*-*csal1570*), and the succinate dehydrogenase (complex II) were induced, whereas the *csal3132* gene, which encodes one of the subunits of the proton-pumping NADH dehydrogenase NDH-I was repressed. Genes for the F0F1 ATP synthase were also induced between 1.80- and 2.36-fold (**Supplementary Table S9**).

Taken together, these findings suggest that *C. salexigens* transcriptionally responds to osmotic and heat stress by modulating the components of the respiratory chain, along with additional systems involved in the generation of H^+^ and/or Na^+^ gradients. Thus, at low salinity the genes encoding the non-proton pumping NADH quinone dehydrogenase (NAD-II) are induced, and quinones could be directly oxidized by the cytochrome *bo*_3_/bd terminal oxidase. This NAD-II might be totally or partially replaced at high salinity with the proton-pumping- NADH dehydrogenase (NAD-I). At high salinity, the ATP synthase genes were also induced, suggesting that in these conditions more energy is necessary for ATP-dependent processes such as compatible solute and flagellum synthesis, and solute (osmoprotectants or nutrients) transport. Interestingly, when temperature was raised at high salinity, further induction of the genes for the ATP synthase and the Na^+^-pumping NADH:quinone oxidoreductase (Na^+^-NQR) was observed. In other bacteria, including marine and pathogenic representatives, Na^+^-NQR is a primary sodium pump, which specifically expels Na^+^ across the cytoplasmic membrane, generating a sodium motive force (SMF) that provides energy for a number of key physiological functions, including rotation of the flagella, uptake of nutrients or ion homeostasis (Barquera, 2014). NQR has been reported to be activated by high Na^+^ concentration (Juárez et al., 2011), but transcriptional activation by temperature had not been reported so far. As temperature could alter H^+^ as well as Na^+^ gradients, probably the induction of a NQR oxidoreductase is needed or preferred to support ATP synthesis and maintain Na^+^ gradients at high temperature.

The contribution of the three respiratory NADH dehydrogenases found in *C. salexigens* genome to the osmo- and heat-stress response needs to be experimentally tested. This availability of three types of NADH dehydrogenases is not common in the prokaryotic world. It may constitute an evolutionary advantage of microorganisms which are facing environmental fluctuations, such as the broad-salinity range bacterium *C. salexigens*.

### Determination of Intracellular Na^+^ Content and Effect of Disturbance of H^+^ and Na^+^ Gradients in Growth and Ectoine Production

In other bacteria, the *in vitro* activity of the enzymes involved in the synthesis of ectoines is influenced by NaCl. Thus, in *H. elongata* (Ono et al., 1999) and the marine bacterium *Sphingopyxis alaskensis* (Widderich et al., 2016), ectoine synthase was reported to increment its activity in response to the NaCl concentration of the assay buffer. On the contrary, the *in vitro* activity of the *S. alaskensis* ectoine hydroxylase was inhibited by NaCl (Widderich et al., 2014). These results suggested that the Na^+^ intracellular content, resulting from entrance versus efflux of NaCl under different salinity conditions, could affect the *in vivo* enzymatic activity, and consequently ectoine synthesis, in these microorganisms. This led us to investigate the transcriptional response of the mechanisms involved in Na^+^ homeostasis.

Genes encoding sodium solute symporters (i.e., *csal0028*, *csal0400*, *csal2891*, and *csal3274*), and secondary Na^+^/H^+^ pumps such as Na^+^/H^+^ antiporters (i.e., *csal1184*, *csal3210*, and *csal2318*) were overexpressed at high salinity (**Supplementary Table S9**). As mentioned before, genes encoding components of the primary sodium-pumping NADH:ubiquinone oxidoreductase (Na^+^-NQR; *csal1569*-*csal1570*) were induced by high temperature at high salinity. In contrast, genes for other Na^+^/H^+^ antiporters (i.e., *csal0898*-*csal0903*) were repressed at high salinity plus high temperature (**Supplementary Table S9**).

**FIGURE 3:**
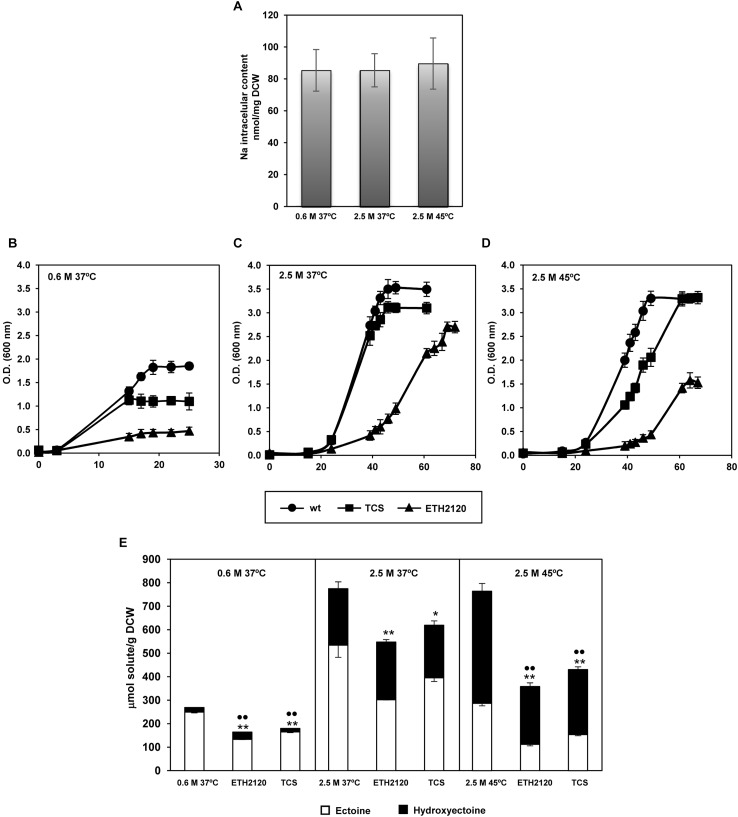
Sodium homeostasis and influence of Na^+^ and H^+^ ionophores in growth and synthesis of ectoines depending on salinity and temperature. **(A)** Intracellular sodium content quantified by inductively coupled plasma optical emission spectrometry (ICP-OES) at low and high salinity, and high salinity plus high temperature. Aliquots of cells grown at mid-late exponential were taken to quantify intracellular content of sodium. **(B–E)** Effect of proton- and sodium-specific ionophores on *C. salexigens* growth and ectoine content under the same conditions as above. *C. salexigens* was grown in the presence of 0.1 μM of tetrachlorosalicylanilide (TCS, protonophore) or 30 μM ETH2120 (sodium-specific ionophore) at 0.6 M NaCl 37°C **(B)**, 2.5 M NaCl 37°C **(C)** and 2.5 M NaCl 45°C **(D)**. Intracellular ectoine and hydroxyectoine content was quantified by LC-MS **(E)**. The values are the averages and standard deviations of three replicates for each condition in two independent experiments. According to Student’s *t*-test, significant differences compared with the control (no ionophore treatment) were showed by two (*p*-value ≤ 0.05) or one (*p-*value ≤ 0.1) asterisks (ectoine) or spots (hydroxyectoine).

The above differential expression of genes responsible for sodium entrance and extrusion, led us to measure *C. salexigens* Na^+^ intracellular content. As shown in **Figure 3A**, intracellular Na^+^ content remained constant (85–90 nmol/mg dry weight), regardless of salinity and temperature. These results suggested that under *in vivo* conditions, synthesis of ectoines in *C. salexigens* at high salinity might not primarily rely on enzymatic activation by Na^+^. This would also indicate that in hyperosmotic conditions Na^+^ gradients are higher and maintained by activated efflux systems, as suggested by the transcriptional response observed. This maintenance of the intracellular Na^+^ levels was not observed in other moderately halophilic bacteria. For example, in the Gram-positive *Tetragenococcus* *halophile*, an increment of intracellular Na^+^ concentration with salinity (from approximately 150 to 2000 nmol/mg DCW from 0.5 to 2 M of NaCl) was observed (Robert et al., 2000). The same behavior was also reported in the anaerobic bacterium *Haloanaerobium praevalens* (Oren, 1986). On the other hand, the intracellular sodium concentration decreased at high salinity in *V. costicola*. However, the latter has to be taken with caution because the differences observed are of doubtful statistical significance as stated by the authors (Gilboa et al., 1991). Altogether, these findings suggest a different regulation of Na^+^ homeostasis in *C. salexigens* and other halophilic bacteria.

On the other hand, the pattern variation in the transcriptional response of H^+^- and/or Na^+^-gradient generators suggested that *C. salexigens* could preferentially rely on PMF (Proton Motive Force) (H^+^ gradient) or SMF (Sodium Motive Force) (Na^+^ gradient) as a source of energy, depending on the salinity and temperature of the external medium. In addition, the synthesis of ectoines is a highly ATP-demanding process (Oren, 1999) that may depend on H^+^- (PMF) and/or Na^+^-(SMF) gradients. In particular, the TeaABC-mediated recovery of ectoines in *H. elongata* uses Na^+^ instead of ATP (Grammann et al., 2002), and therefore would depend more on Na^+^-gradients.

In a preliminary attempt to test the contribution of PMF and SMF to *C. salexigens* growth and ectoine production under osmotic and heat stress, we measured growth and content of ectoines when Na^+^ and H^+^ gradients were disturbed by specific ionophores. Cells were grown at the three experimental conditions as for RNA-seq, in the absence or presence of the Na^+^-specific ionophore ETH2120, or the H^+^- specific ionophore 3,3^′^,4^′^,5-tetrachlorosalicylanilide (TCS). As shown in **Figures 3B–D**, both uncouplers caused an impaired growth in all experimental conditions tested. In the case of TCS, the impact on growth was higher at low salinity, with only a slightly influence in growth at high salinity (independently of temperature) observed. Regarding ETH2120, it clearly produced a delayed growth in all conditions tested, but especially at low salinity. These findings highlight the importance of both PMF and (especially) SMF for *C. salexigens* life, even at low salinity. They also point out differences in the magnitude of Na^+^ and H^+^ gradients depending on the stress condition. Nevertheless, further experiments are needed to get insights into the involvement of PMF and SMF in *C. salexigens* growth under osmotic and heat stress conditions.

Finally, we investigated how disturbance of Na^+^- and H^+^- gradients influences production of ectoines. At 37°C (with low or high salinity), alteration of the Na^+^ and H^+^ gradients resulted in a reduction of the ectoine content (46.54 ± 2.19% and 43.45 ± 9.97% for ETH2120, and 33.78 ± 3.08 and 26.02 ± 12.7% for TCS, at low and high salinity, respectively) (**Figure 3E**). None of the ionophores had a strong effect in the hydroxyectoine content at 37°C, regardless salinity. Finally, under conditions of high salinity and high temperature, the two uncouplers caused around 45–60% reduction of ectoine (61 ± 6.01% for ETH2120 and 46.32 ± 5.8% for TCS) and hydroxyectoine (48.36 ± 10.2% for ETH2120 and 42.1 ± 9.38% for TCS) content (**Figure 3E**). These results showed the dependence of production of ectoines on both Na^+^ and H^+^ gradients, but differences were found depending on the solute, the salinity, and temperature conditions.

### Motility Under Osmotic and Heat Stress, and Its Dependence on Na^+^-Gradients

Remarkably, flagellum genes presented the highest differential expression values found in this study. A considerable number of genes responsible for the synthesis and regulation of the flagellum were induced by high salinity (1.84- to 6.40-fold), and most of them were strongly induced by high temperature (4- to 33.8-fold) (**Supplementary Table S10**). This transcriptional pattern suggested that cell motility could be stimulated at high salinity, and especially at high temperature.

**FIGURE 4:**
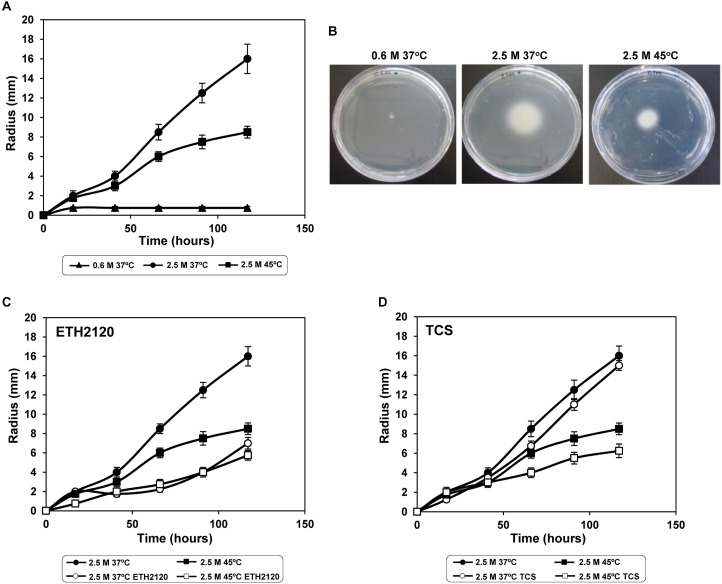
*C. salexigens* swarming tests under osmotic and heat stresses, and influence by Na^+^ and H^+^ ionophores. To evaluate motility of *C. salexigens* in M63 semisolid agar, 3 μl aliquots of mid-exponential cells were inoculated near the center of M63 swarming plates containing 0.35% of agar and 20 mM glucose, at low salinity (0.6 M NaCl, 37°C), high salinity (2.5 M NaCl, 37°C) and high salinity plus high temperature (2.5 M NaCl, 45°C). In panel **(A)**, migrating radii are plotted against time (h) at the three experimental conditions. Panel **(B)** illustrates migration in M63 semisolid agar plates at 90 h of incubation. The swarming tests were also performed in the presence of 30 μM of the sodium ionophore ETH2120 **(C)**, or 0.1 μM of the protonophore tetrachlorosalicylanilide (TCS) **(D)**. Radii of migration were measured from three independent repetitions, and average values and SD are shown.

To experimentally confirm this hypothesis, we performed swarming tests in the three experimental conditions used for RNA-seq. As expected, no cell motility was observed at low salinity, and motility was induced by high salinity (**Figures 4A,B**). In agreement with the observed transcriptional pattern, motility was salinity-induced, as a time-dependent increase of the motility zone was observed at 2.5 M NaCl (**Figure 4A**). Specifically, the radii of the motility zones were 0.75 ± 0.1 mm and 12.5 ± 1 mm at low and high salinity, respectively, after 90 h incubation (**Figure 4A**). This motility pattern was also observed in halotolerant bacteria, such as *V. salmonicida* (Karlsen et al., 2008) or *Tistlia consotensis* (Rubiano-Labrador et al., 2015), and in the moderate halophile *Halobacillus halophilus* (Roessler et al., 2000), but not in the moderate halophile *J. malaysiensis* (Yaakop et al., 2016). In the case of *V. salmonicida* and *H. halophilus* a transcriptional induction of flagellum genes by salinity was also detected (Roessler et al., 2000; Karlsen et al., 2008). The NaCl-dependent induction of *C. salexigens* motility suggested that it might have a Na^+^-driven flagellar motor. This hypothesis was addressed below.

In spite of the strong transcriptional induction of flagellum genes, *C. salexigens* motility was reduced at high temperature (**Figures 4A,B**). The radius of the mobility zone after 90 h incubation at 2.5 M NaCl and 45°C was 7.5 ± 0.7 mm, and the migration rate decreased from 0.15 ± 0.01 mm/h (at 2.5 M NaCl and 37°C) to 0.09 ± 0.002 mm/h (at 2.5 M NaCl and 45°C) (**Figures 4A,B**). This was not the case of *Burkholderia thailandensis* (Peano et al., 2014) or *Pseudomonas syringae* (Hocckett et al., 2013), where increment of temperature led to both transcriptional repression of flagellum genes and reduction of motility.

To preliminary investigate if *C. salexigens* flagellar motor is dependent on Na^+^ and/or H^+^ gradients, we performed the swarming tests in the presence of Na^+^- or H^+^- specific ionophores. As shown in **Figures 4C,D**, motility in both conditions was mainly impaired when the Na^+^ gradient was altered, whereas alteration of the H^+^ gradient had no significant impact on motility. This result suggests that *C. salexigens* flagellar motor could be mainly Na^+^-driven, regardless of temperature. This dependence of Na^+^ gradient, which provides energy for polar flagella, has been demonstrated in marine species such as *Vibrio* (Atsumi et al., 1992; Yorimitsu and Homma, 2001).

Our results are consistent with the fact that in *C. salexigens* the intracellular Na^+^ level is constant at different salinities. This implies that the Na^+^ gradient is higher at 2.5 M than at 0.6 M NaCl. Therefore, it could be used for flagellar rotation. This could also explain the reduction of motility at high temperature, which might be due to a reduction of the Na^+^ gradient produced by an alteration of ion membrane permeability.

### Iron Homeostasis Mechanisms: Connection With Osmo- and Thermo-Adaptation, and Synthesis of Ectoines

Next we focused on the correlation of iron homeostasis with the osmo- and heat-stress responses, and their connection with the synthesis of ectoines. It is because network clustering analysis revealed different patterns of expression of proteins related to iron homeostasis in response to salinity and temperature (**Supplementary Table S11** and **Supplementary Figure S3**). In contrast to *Bacillus subtilis*, for which high salinity imposes iron limitation (Hoffmann et al., 2002), the iron demand of *C. salexigens* was higher at low salinity, which was correlated with a higher siderophore production under this condition (Argandoña et al., 2010). In fact, genes encoding ABC-type transport systems for siderophores (*csal1449*, *csal2549, csal2702*, *csal3089*, and *csal3091*) and the isochorismate-pyruvate hydrolase (*csal1779* and *csal2494*), responsible for initiating siderophore biosynthesis, were induced at low salinity. In addition, genes encoding iron-chelating agents like bacterioferritin (*csal2900*) and the Dps ferritin (*csal2953*) were induced at low salinity (**Supplementary Table S11**).

Remarkably, a different set of transport systems related to iron homeostasis was induced at high salinity, including genes for other siderophore-iron transport system (*csal1040-1045*), several TonB receptors (*csal3258*, *csal3182*, and *csal2539*), and a different siderophore biosynthesis and transport cluster (*csal1053-1056*). In addition, high salinity resulted in induction and repression of the genes *csal1052* and *csal1098*, respectively, encoding orthologs of the sigma factor FecI, which is a transcriptional activator of genes of the siderophore and iron homeostasis regulon (Angerer et al., 1995). Interestingly, the ferric-iron reductase *csal3257* was strongly induced at high salinity, suggesting a role in controlling iron homeostasis under this condition (**Supplementary Table S11**). At high salinity and high temperature, a strong induction of genes encoding a different ABC-type iron-siderophore transport system (*csal2699-csal2702*) was observed, together with repression of the salt-induced siderophore biosynthesis and transport cluster (*csal1053-1056*), and the periplasmic proteins from the siderophore-independent iron uptake system EfeB and EfeO (*csal3311* and *csal3312*) (**Supplementary Table S11**).

**FIGURE 5:**
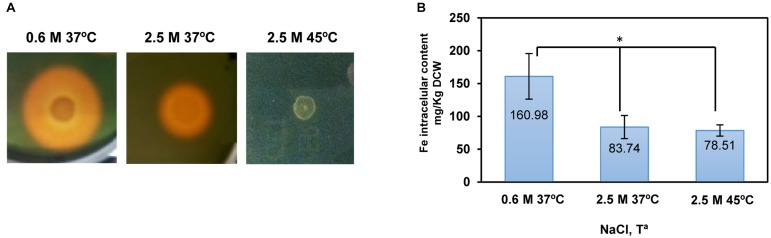
Influence of salinity and temperature on siderophore production and intracellular iron content in *C. salexigens*. **(A)** Siderophore production assay was performed in M63 modified medium as described previously (Argandoña et al., 2010) with different concentration of NaCl and temperatures (0.6 or 2.5 M, at 37 or 45°C). The diameter of the orange halo is directly related to siderophore production. **(B)** The intracellular iron content of cells grown in M63 with 0.6 M NaCl at 37°C, 2.5 M NaCl at 37°C, or 2.5 M NaCl at 45°C, was determined by inductively coupled plasma optical emission spectrometry (ICP-OES). Aliquots of cells grown at mid-late exponential were taken to quantify intracellular iron content. The values are the average and the standard deviations of three replicates for each condition in two independent experiments. According to Student’s *t*-test, significant differences (*p*-value ≤ 0.05) compared with 0.6 M NaCl 37°C were showed by an asterisk.

In an attempt to compare the gene expression pattern with the iron homeostasis mechanisms in response to salt and heat stress, we investigated *C. salexigens* siderophore production and intracellular iron content under the same experimental conditions as for RNA-seq. Both a higher siderophore production (which agrees with previous results; Argandoña et al., 2010), and a higher intracellular iron content, were observed at low salinity (**Figure 5**). Both findings correlated well with the transcriptional response. As mentioned before, the higher demand for iron (Argandoña et al., 2010) and concomitant higher iron content (this work), could be one of the causes of the secondary oxidative stress response observed (at the transcriptional level) at low salinity. Indeed, the hydroxyl radical (HO^.^) produced in the Fenton reaction between H_2_O_2_ and the intracellular pool of labile iron could cause protein and DNA damage (Henle et al., 1999), triggering the activation of secondary mechanisms that repair or exert protection over these molecules (see above).

Transcriptional induction of a different set of siderophore biosynthesis and transport genes by salinity was not associated with a higher siderophore production and intracellular iron level, as both siderophore production and intracellular iron content were lower at high salinity (**Figure 5**). This fact suggests that an increased level of transcript does not always correlates with a higher production of the coded function. It is possible that *C. salexigens* synthesizes at least two types of differently regulated siderophores, depending on salinity, which are indistinguishable by the siderophore production assay. On the other hand, metabolic requirements may affect siderophore production at high salinity. In this respect, the ammonium uptake rate in *C. salexigens* is practically constant regardless of salinity, while the total protein content is reduced with salinity (Argandoña et al., 2010; Pastor et al., 2013). As ectoines are nitrogen-containing molecules, at high salinity, *C. salexigens* cells balance their nitrogen content and ectoines are accumulated at the expense of proteins (Pastor et al., 2013). In addition, siderophores, which are very often based on amino acids and their derivatives, also require nitrogen for their synthesis. In fact, histidine has been suggested to be involved in siderophore biosynthesis in *C. salexigens* (Argandoña et al., 2010). Therefore, it is plausible that at high salinity nitrogen channeling to ectoines might reduce not only the synthesis of proteins but also of other nitrogen-containing molecules, such as siderophores.

Interestingly, the intracellular iron content did not change in response to temperature, despite no siderophore secretion was observed (**Figure 5**). In addition, siderophore synthesis genes were repressed at 45°C (**Supplementary Table S11**). These findings suggest that in *C. salexigens* siderophore-independent iron uptake systems might be responsible for maintaining iron homeostasis at high temperature, where iron solubility is lower (Liu and Millero, 2002).

Both ectoine synthase and ectoine hydroxylase (responsible for the synthesis of ectoine and hydroxyectoine, respectively) are iron-dependent enzymes (Reuter et al., 2010; Widderich et al., 2014). The relationship between iron homeostasis and synthesis of ectoines in *C. salexigens* has been reported previously. Thus, an excess of iron negatively modulated the activity of a transcriptional fusion between the promoter of the ectoine synthesis gene *ectA* and the reporter gene *lacZ* in an *E. coli* background (Calderón et al., 2004). In addition, excess of iron increased the cytoplasmic hydroxyectoine content of *C. salexigens* cells grown at high salinity, at the expense of ectoine. These findings suggested that hydroxyectoine might confer better protection against the iron-induced oxidative stress than ectoine (Argandoña et al., 2010). Moreover, the iron homeostasis regulator Fur was found to up-regulate the ectoine synthesis genes *ectABC* (Argandoña et al., 2010).

**FIGURE 6:**
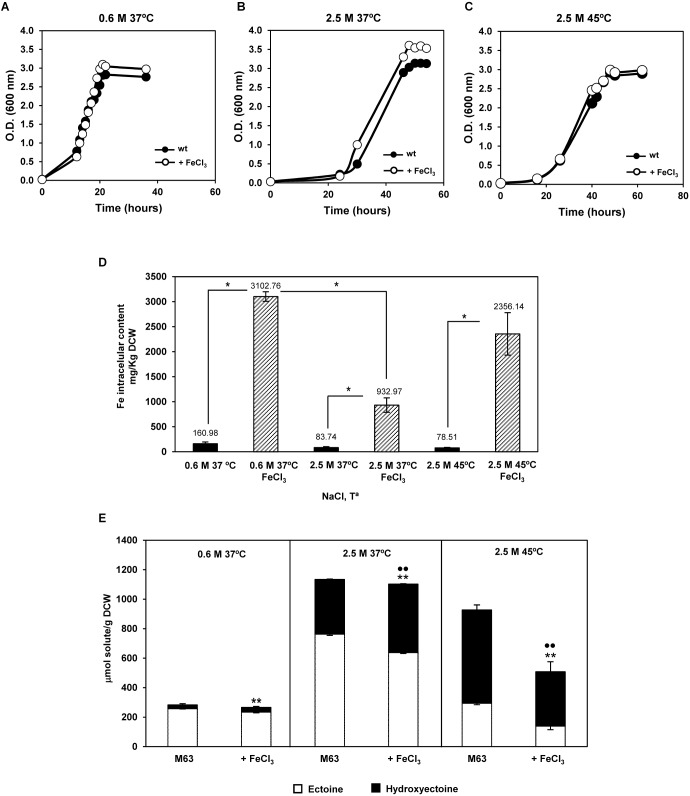
Influence of external iron on growth and production of ectoines by *C. salexigens*. Cells were grown in glucose M63 minimal medium, in the presence or absence of 50 μM FeCl_3_, at 0.6 M NaCl and 37°C **(A)**, 2.5 M NaCl and 37°C **(B)**, and 2.5 M NaCl and 45°C **(C)**. Aliquots of cells grown at mid-late exponential were taken to quantify intracellular content of iron by inductively coupled plasma optical emission spectrometry (ICP-OES). According to Student’s *t*-test, significant differences (*p*-value ≤ 0.05) compared with the control were showed by an asterisk **(D)**. Ectoine and hydroxyectoine content was quantified by LC-MS **(E)**. The values are the average and the standard deviations of three replicates for each condition in two independent experiments are showed. According to Student’s *t*-test, significant differences (*p*-value ≤ 0.05) compared with the control (no added FeCl_3_) were showed by two asterisks (ectoine) or spots (hydroxyectoine).

The above antecedents led us to test if changes in the iron external supply might affect growth, intracellular iron content, and/or production of ectoines, under the three conditions as for RNA-seq. Iron supply did not significantly alter growth at any of the three experimental conditions tested (**Figures 6A–C**), though it strongly incremented the intracellular iron content (**Figure 6D**). In addition, external iron did not influence accumulation of ectoines at low salinity, but increased hydroxyectoine accumulation by 20% at high salinity and 37°C, as already reported (Argandoña et al., 2010). On the other hand, an excess of iron reduced the ectoine and hydroxyectoine intracellular content by 50% at high salinity plus high temperature (**Figure 6E**), despite growth was not affected (**Figure 6C**). This finding was somehow surprising, as hydroxyectoine is involved in *C. salexigens* thermoprotection (García-Estepa et al., 2006). One hypothesis is that at high temperature a thermoprotective, iron-dependent mechanism could be induced, and compensate this reduction in hydroxyectoine accumulation.

The above data suggest that the underlying mechanisms responsible for iron homeostasis and its connection with synthesis of ectoines in *C. salexigens* are complex and diverse, and affected by the salinity and temperature of the external medium. Iron homeostasis regulation, and its relation to synthesis of ectoines and thermoadaptation, deserves further experimental analysis.

## Concluding Remarks and Open Questions

In this work, we addressed the global transcriptional response of the halophilic bacterium *C. salexigens* to osmotic and heat stress, in conditions of low and high ectoine and hydroxyectoine production. This global analysis was complemented with physiological assays, in order to give experimental support to our main conclusions. Our findings revealed that both stresses have a significant impact on *C. salexigens* global physiology. Apart from specific osmo- and heat-stress response mechanisms such as those related to compatible solute metabolism (uptake, synthesis and degradation), most relevant adaptation systems were related to oxidative and protein folding stress responses, respiratory chain and related components, and ion (sodium and iron) homeostasis. Interestingly, this study generates a number of open questions that would be interesting to address in the future: (i) the role and regulation of the putative ectoine synthesis/degradation/recycling cycle in *C. salexigens*, as well as the suggested heat-induced post-transcriptional regulation of hydroxyectoine synthesis, (ii) the possible involvement of hydroxyectoine in protecting cells against the salt-induced oxidative stress at high temperature, (iii) the physiological basis for the protein-folding-stress faced by *C. salexigens* at low salinity, (iv) the variation in the composition of *C. salexigens* respiratory chain, the contribution of the three respiratory NADH dehydrogenases, and H^+^/Na^+^ gradients, to osmotic and heat stress adaptation, and their influence in synthesis of ectoines, and (v) the iron homeostasis mechanisms and their regulation, especially at high temperature, and its connection with production of ectoines or other metabolic requirements (i.e., for nitrogen). Finally, one of the most intriguing questions remaining is the basis of *C. salexigens* halophilism. The finding that alteration in Na^+^ gradients (and therefore SMF) causes a growth arrestment especially at low salinity, opens the question about which Na^+^-dependent processes are necessary for *C. salexigens* growth at lower salt limits.

## Author Contributions

MA and CV conceived and supervised the study. MS and MA performed the RNA-seq experiments and MS the bioinformatics analysis. MS, FP, MA, and EN developed experimental phenotypic analysis. MS, MA, LC, JN, and CV were involved in the analysis and discussion of results. MS, MA, and CV drafted the manuscript. All authors revised and approved the final manuscript.

## Conflict of Interest Statement

The authors declare that the research was conducted in the absence of any commercial or financial relationships that could be construed as a potential conflict of interest.

## Footnotes

1 http://www-huber.embl.de/users/anders/HTSeq

2 http://www.r-project.org/

3 http://www.ncbi.nlm.nih.gov/COG/; Date of last access: 12^th^ of January 2018

4 http://david.abcc.ncifcrf.gov/; Date of last access: 12^th^ of January 2018
