# Supplementary material for: Quantitative RNA-seq Analysis Unveils Osmotic and Thermal Adaptation Mechanisms Relevant for Ectoine Production in Chromohalobacter salexigens
Source: Front Microbiol. 2018 Aug 13;9:1845. doi: 10.3389/fmicb.2018.01845 (PMC6104435; doi:10.3389/fmicb.2018.01845)
Supplement: Supplementary file 1 [file Presentation_1.PDF]

Supplementary Material

Quantitative RNA-seq analysis unveils osmotic and thermal adaptation mechanisms relevant for ectoine production in *Chromohalobacter salexigens*

Manuel Salvador[a,b], Montserrat Argandoña[a], Emilia Naranjo[a], Francine Piubeli[a], Joaquín J. Nieto[a], Laszlo N. Csonka[c], and Carmen Vargas[a*]

[a]*Department of Microbiology and Parasitology, Faculty of Pharmacy, University of Sevilla, Sevilla, Spain.*

[b]*Faculty of Health and Medical Sciences, University of Surrey, Guildford, UK*

[c]*Department of Biological Sciences, Purdue University, West Lafayette, IN 47907-2064, USA.*

*** Correspondence:** Corresponding Author: cvargas@us.es

1 Supplementary Figures and Tables

1.1 Supplementary Figures

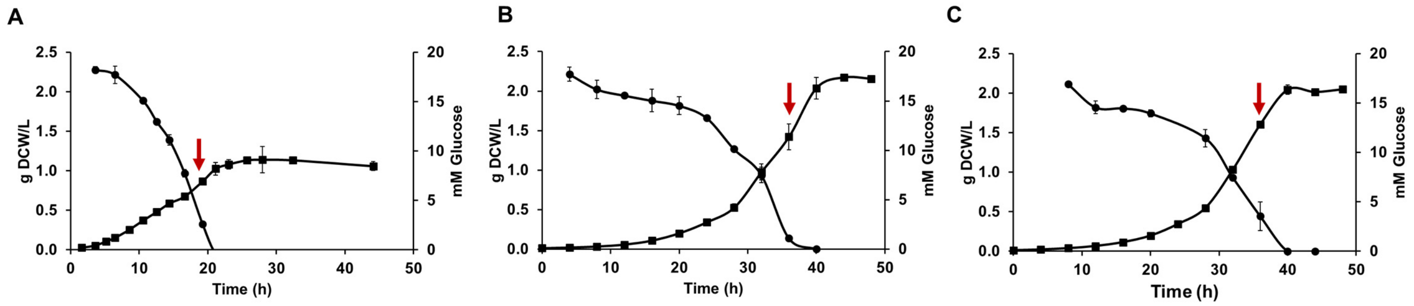

Supplementary Figure S1. Growth and glucose consumption of *C. salexigens* at different salinities and temperatures. Biomass (g Dry Cell Weight (DCW)/L) (squares) and glucose concentration (mM) (circles) in the media during growth of *C. salexigens* in M63 glucose minimal medium (20 mM) at 0.6 M NaCl and 37ºC (A), 2.5 M NaCl and 37ºC (B) and 2.5 M NaCl and 45ºC (C). Points of samples collection for RNA-seq experiments were indicated with a red arrow.

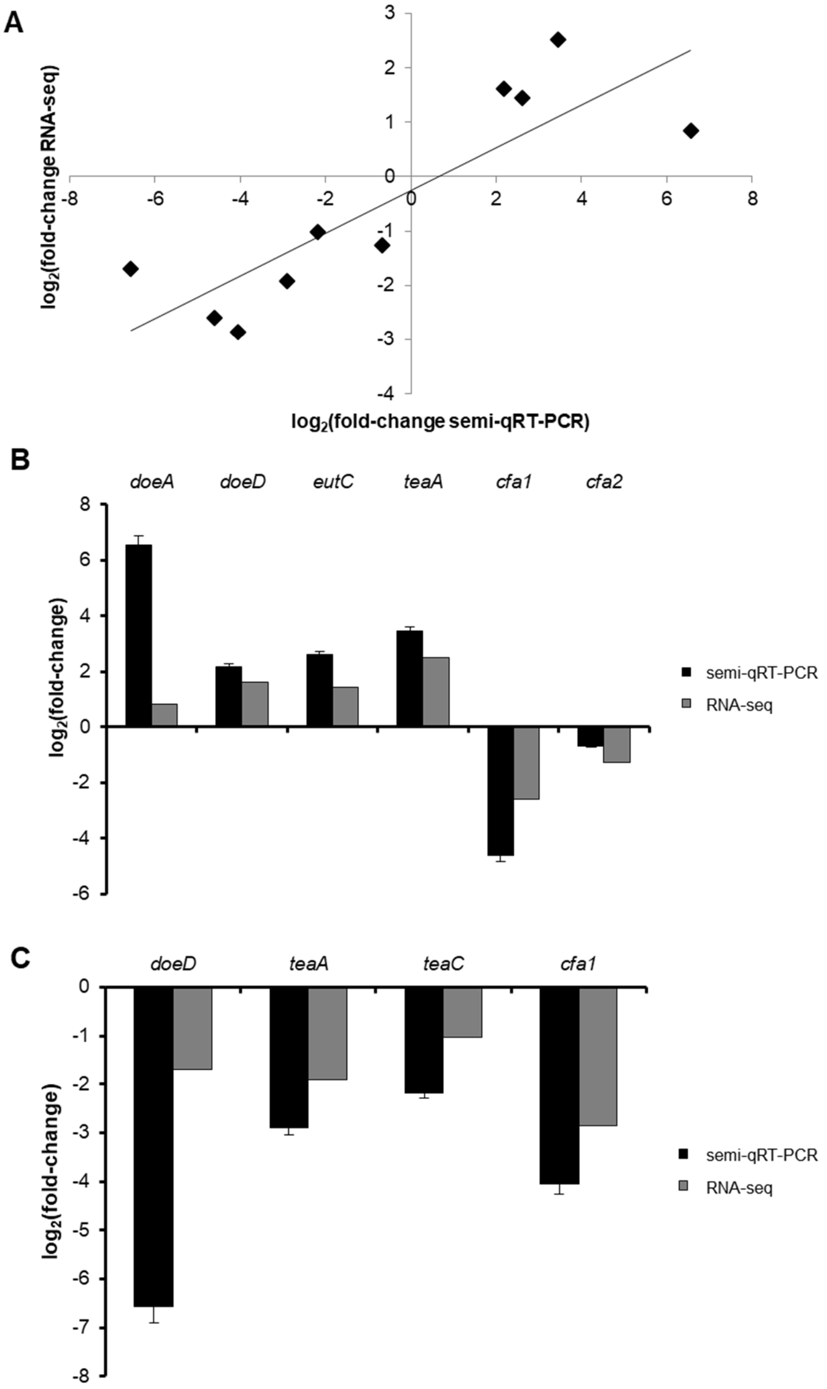

Supplementary Figure S2. Semi-qRT-PCR validation of differentially expressed genes. Correlation of the log_2 of the fold-change of the expression levels of genes determined by semi-qRT-PCR and by RNA-seq (A). The log_2 of the fold difference in gene expression as determined by semi-qRT-PCR (black columns) is plotted next to the results obtained in RNA-seq analysis (grey columns) for cultures grown at 2.5 M NaCl / 0.6 M NaCl at 37°C (B) and at 2.5 M NaCl at 37°C / 2.5 M NaCl at 45°C (C). The results of semi-qRT-PCR are expressed as means ± SD for three separate experiments performed in duplicate

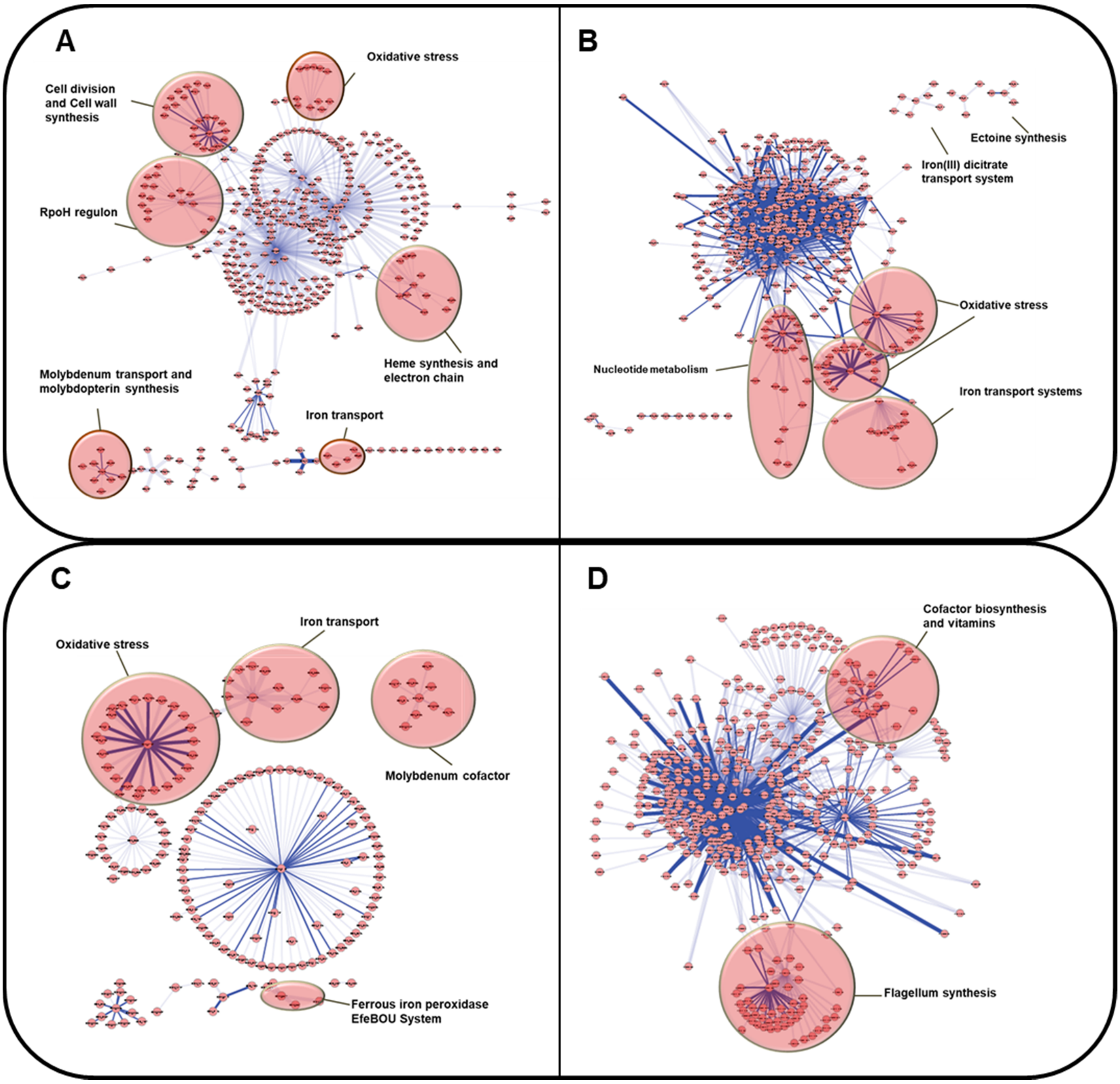

Supplementary Figure S3. Functional clustering network analysis of *C. salexigens* genes differentially induced at low salinity (A) and high salinity (B) and genes repressed (C) and induced (D) by high temperature at high salinity. Clusters of nodes of special interest and/or whose members belong to the same functional group (as found overrepresented in the DAVID analysis) are highlighted by red circles.

1.2. Supplementary Tables

Table S1. Primers used in semi-qRT-PCR experiments to validate SOLID RNAseq results.

Primer	Sequence 5'-3'	Amplified gene
cfa1_Fw	TATCTGGATACCTATCTG	*cfa1* (*csal0460*)
cfa1_Rv	AGATGTAGCGCTTGATGA	
cfa2_Fw	ATTTCGGCTTCAGGACTATCG	*cfa2* (*csal1480*)
cfa2_Rv	GGGCGACCTCCATGTACT	
doeA_Fw	TGGCACAACGGTATCATC	*doeA (csal2732)*
doeA_Rv	ACCAGTTGACCAGCAAAT	
doeD_Fw	CACGACCACTTCGACCTG	*doeD (csal2724)*
doeD_Rv	GAAAACTCGCGCTCGCTCATG	
eutC_Fw	GCTGATGATGGTGTTCTCG	*eutC* (*csal2722*)
eutC_Rv	CGAGACATCGACACCGT	
teaA_Fw	ATGCGAAATATCGTCCTGAGTATC	*teaA (csal3140)*
teaA_Rv	GCATAGCCGTACTGCACGCT	
teaC_Fw	CACGCTGTTCATGATGTTCAC	*teaC (csal3139)*
teaC_Rv	TACCCAACTGCCGATACCGTT	

Table S2. Summary of SOLiD RNA-seq results and mapping statistics

Growth Condition Sample	Millions of Reads	% Reads Aligned to Genome	% Reads Aligned in Pairs
0.6 M NaCl 37°C (A)	85.97	75.28	53.58
0.6 M NaCl 37°C (B)	93.69	73.07	49.56
0.6 M NaCl 37°C (C)	93.08	75.34	54.61
2.5 M NaCl 37°C (A)	70.56	73.48	47.03
2.5 M NaCl 37°C (B)	83.59	75.00	48.36
2.5 M NaCl 37°C (C)	67.96	75.79	52.54
2.5 M NaCl 45°C (A)	54.58	74.36	48.67
2.5 M NaCl 45°C (B)	72.02	74.19	47.75
2.5 M NaCl 45°C (C)	73.64	74.56	48.76

Table S3. Reproducibility at the expression level between replicates

PEARSON CORRELATION COEFFICIENT		0.6 M 37°C			2.5 M 37°C			2.5 M 45°C		
		A	B	C	A	B	C	A	B	C
0.6 M 37°C	A	1.00								
	B	0.96	1.00							
	C	0.94	0.97	1.00						
2.5 M 37°C	A	0.67	0.70	0.71	1.00					
	B	0.66	0.69	0.69	0.94	1.00				
	C	0.69	0.71	0.72	0.87	0.94	1.00			
2.5 M 45°C	A	0.60	0.62	0.62	0.79	0.82	0.77	1.00		
	B	0.61	0.65	0.65	0.84	0.86	0.81	0.92	1.00	
	C	0.65	0.68	0.68	0.79	0.81	0.82	0.88	0.95	1.00

Table S4. Enriched ontology terms provided by DAVID when studying the differentially expressed genes at low salinity and high salinity

GO Number	Term	%	*p*-value
**Genes overexpressed al low salinity 37ºC**			
GO:0033554	cellular response to stress	2.66	3.71E-02
GO:0006732	coenzyme metabolic process	3.61	5.19E-02
GO:0051186	cofactor metabolic process	4.57	5.43E-02
GO:0006281	DNA repair	2.47	4.53E-02
GO:0055114	oxidation reduction	9.71	3.23E-02
GO:0006457	protein folding	1.33	6.88E-02
GO:0009306	protein secretion	1.90	9.96E-03
GO:0051252	regulation of RNA metabolic process	8.19	8.37E-02
GO:0006355	regulation of transcription, DNA-dependent	8.19	7.92E-02
GO:0006974	response to DNA damage stimulus	2.47	4.53E-02
GO:0046903	secretion	1.90	9.96E-03
GO:0032940	secretion by cell	1.90	9.96E-03
**Genes overexpressed al high salinity 37ºC**			
GO:0006754	ATP biosynthetic process	1.38	6.02E-03
GO:0046034	ATP metabolic process	1.38	6.02E-03
GO:0015986	ATP synthesis coupled proton transport	1.18	1.33E-02
GO:0007610	behavior	1.38	8.52E-02
GO:0008643	carbohydrate transport	1.18	5.29E-02
GO:0006812	cation transport	3.55	5.84E-02
GO:0006928	cell motion	1.38	8.52E-02
GO:0006935	chemotaxis	1.38	8.52E-02
GO:0009262	deoxyribonucleotide metabolic process	0.78	9.57E-02
GO:0015985	energy coupled proton transport, down electrochemical gradient	1.18	1.33E-02
GO:0006818	hydrogen transport	1.18	2.89E-02
GO:0034220	ion transmembrane transport	1.38	1.92E-02
GO:0007626	locomotory behavior	1.38	8.52E-02
GO:0015672	monovalent inorganic cation transport	2.56	8.74E-02
GO:0009142	nucleoside triphosphate biosynthetic process	1.57	4.30E-03
GO:0009141	nucleoside triphosphate metabolic process	1.57	9.24E-03
GO:0006119	oxidative phosphorylation	1.97	9.25E-04
GO:0016310	phosphorylation	2.36	6.53E-02
GO:0042777	plasma membrane ATP synthesis coupled proton transport	0.78	2.38E-02
GO:0015992	proton transport	1.18	2.89E-02
GO:0009145	purine nucleoside triphosphate biosynthetic process	1.58	1.67E-03
GO:0009144	purine nucleoside triphosphate metabolic process	1.58	2.75E-03
GO:0006164	purine nucleotide biosynthetic process	1.78	5.64E-02
GO:0006163	purine nucleotide metabolic process	1.78	7.85E-02
GO:0009206	purine ribonucleoside triphosphate biosynthetic process	1.58	1.67E-03
GO:0009205	purine ribonucleoside triphosphate metabolic process	1.58	2.75E-03
GO:0009152	purine ribonucleotide biosynthetic process	1.58	6.92E-02
GO:0009150	purine ribonucleotide metabolic process	1.58	8.24E-02
GO:0009201	ribonucleoside triphosphate biosynthetic process	1.58	1.67E-03
GO:0009199	ribonucleoside triphosphate metabolic process	1.58	2.75E-03
GO:0042330	taxis	1.38	8.52E-02
GO:0006412	translation	5.91	1.70E-04
GO:0055085	transmembrane transport	1.97	2.11E-02

Table S5. Enriched ontology terms provided by DAVID when studying the differentially expressed genes at high salinity and high salinity plus temperature

GO Number	Term	%	*p*-value
**Genes overexpressed al high salinity 37ºC**			
GO:0032324	molybdopterin cofactor biosynthetic process	1.1	1.85E-02
GO:0043545	molybdopterin cofactor metabolic process	1.1	1.85E-02
GO:0006777	Mo-molybdopterin cofactor biosynthetic process	1.1	1.85E-02
GO:0019720	Mo-molybdopterin cofactor metabolic process	1.1	1.85E-02
GO:0055114	oxidation reduction	8.9	6.38E-03
GO:0006817	phosphate transport	0.8	9.51E-02
GO:0051189	prosthetic group metabolic process	1.1	1.85E-02
GO:0042559	pteridine and derivative biosynthetic process	1.1	4.61E-02
GO:0042558	pteridine and derivative metabolic process	1.1	8.60E-02
**Genes overexpressed al high salinity 45ºC**			
GO:0007610	behavior	5.2	7.56E-09
GO:0048870	cell motility	6.7	1.62E-15
GO:0006928	cell motion	7.1	8.12E-16
GO:0030031	cell projection assembly	3.0	1.48E-05
GO:0030030	cell projection organization	4.5	4.05E-09
GO:0010927	cellular component assembly involved in morphogenesis	1.5	3.16E-03
GO:0032989	cellular component morphogenesis	1.5	1.37E-02
GO:0006935	chemotaxis	5.2	7.56E-09
GO:0001539	ciliary or flagellar motility	6.7	1.62E-15
GO:0009296	flagellum assembly	3.0	1.91E-06
GO:0043064	flagellum organization	4.5	2.74E-10
GO:0009098	leucine biosynthetic process	1.1	7.44E-02
GO:0006551	leucine metabolic process	1.1	7.44E-02
GO:0051674	localization of cell	6.7	1.62E-15
GO:0007626	locomotory behavior	5.2	7.56E-09
GO:0009112	nucleobase metabolic process	1.5	7.91E-02
GO:0042330	taxis	5.2	7.56E-09
GO:0019067	viral assembly, maturation, egress, and release	1.5	3.16E-03
GO:0019069	viral capsid assembly	1.5	3.16E-03
GO:0019058	viral infectious cycle	1.5	3.16E-03
GO:0016032	viral reproduction	1.5	3.16E-03
GO:0022415	viral reproductive process	1.5	3.16E-03
GO:0019068	virion assembly	1.5	3.16E-03

TABLE S6. Differentially expressed genes related to compatible solutes metabolism

Annotation nº and gene name	NCBI ANNOTATION	COG NUMBER	KO ORTHOLOGY NUMBER	fold-change 2.5 M NaCl 37ºC/ 0.6 M NaCl 37ºC	*p*-value	fold-change 2.5 M NaCl 45ºC/ 2.5 M NaCl 37ºC	*p*-value
Osmoprotectant uptake systems							
csal0223	choline transport protein BetT	COG1292M		4.27	5.04E-09		
csal0537	glycine betaine ABC transporter substrate-binding protein	COG2113E				-1.97	2.02E-02
csal1517	glycine betaine ABC transporter substrate-binding protein	COG2113E	K02002	2.73	6.35E-05	-2.12	5.14E-03
csal1701	glycine betaine/choline ABC transporter substrate-binding protein	COG1732M		2.74	2.10E-04		
csal1901	glycine betaine ABC transporter substrate-binding protein	COG2113E	K02002	3.19	2.75E-06	-1.86	2.41E-02
csal2356	glycine betaine ABC transporter substrate-binding protein	COG1732M	K05845			-2.40	1.40E-03
csal2357	glycine betaine ABC transporter substrate-binding protein	COG1732M				-2.71	5.12E-04
csal2645	choline transport protein BetT	COG1292M	K02168	-2.26	2.59E-03	-2.17	7.48E-03
csal2771	glycine betaine ABC transporter substrate-binding protein	COG2113E		13.19	8.81E-23	-2.51	6.42E-04
csal2886	glycine betaine ABC transporter substrate-binding protein	COG2113E	K02002	2.98	2.64E-03		
csal2943	glycine betaine ABC transporter substrate-binding protein	COG1732M	K05845			-1.99	1.31E-02
csal2946	binding-protein-dependent transport system inner membrane protein	COG1174E	K05846	-1.95	1.58E-02		
csal3102	glycine betaine ABC transporter substrate-binding protein	COG2113E	K02002	2.14	2.88E-03	-2.13	4.95E-03
csal3138 teaB	tripartite ATP-independent periplasmic transporter DctQ	COG3090G				-2.89	3.28E-04
csal3139 teaC	TRAP dicarboxylate transporter subunit DctM	COG1593G				-2.03	1.72E-02
csal3140 teaA	TRAP dicarboxylate transporter subunit DctP	COG1638G		5.71	1.07E-12	-3.78	6.89E-08
csal3169	glycine betaine ABC transporter substrate-binding protein	COG2113E				-2.64	2.01E-04
Ectoine metabolism							
csal0542 ectD	ectoine hydroxylase	COG5285Q	K10674	2.79	4.14E-05		
csal0626	aspartate kinase	COG0527E	K00928	2.37	6.06E-04	-1.86	2.52E-02
csal2722 eutC	ectoine utilization protein EutC	COG2423E	K01750	2.71	9.72E-05		
csal2723 eutB	threonine dehydratase	COG1171E	K01754	3.41	1.21E-06		
csal2724 doeD	hypothetical protein	COG0161H	K15785	3.07	8.60E-06	-3.26	3.21E-06
csal2725 doeC	succinate semialdehyde dehydrogenase	COG1012C	K15786	4.68	5.81E-10	-4.02	2.48E-08
csal2731 doeB	succinylglutamate desuccinylase/aspartoacylase	COG3608R	K15784	2.46	4.70E-04	-2.43	8.20E-04
csal2732 doeA	peptidase M24	COG0006E	K15783	1.78	3.10E-02		
csal2770 ybbh2	γ-butyrobetaine,2-oxoglutarate dioxygenase	COG2175Q	K00471	13.15	1.81E-17		
Betaine metabolism							
csal1515	betaine aldehyde dehydrogenase	COG1012C	K00130			1.95	1.57E-02
csal1516	transcriptional regulator BetI	COG1309K	K02167			1.86	3.00E-02
csal1706	betaine aldehyde dehydrogenase	COG1012C	K00130			3.85	1.42E-04
csal2844	betaine aldehyde dehydrogenase	COG1012C	K00130	2.00	1.05E-02		

Table S7. Differentially expressed genes related to oxidative stress response

Annotation nº and gene name	NCBI ANNOTATION	COG NUMBER	KO ORTHOLOGY NUMBER	fold-change 2.5 M NaCl 37ºC/ 0.6 M NaCl 37ºC	*p*-value	fold-change 2.5 M NaCl 45ºC/ 2.5 M NaCl 37ºC	*p*-value
Antioxidant enzymes							
csal0037	OsmC-like protein	COG1764O		4.44	4.22E-09	-2.44	9.15E-04
csal0159 katG	catalase/peroxidase HPI	COG0376P	K03782	2.21	4.85E-03		
csal0179	1-Cys peroxiredoxin	COG0450O		2.12	3.28E-03	-2.14	4.41E-03
csal0321	alkyl hydroperoxide reductase	COG1225O		2.05	7.82E-03		
csal0397	copper/zinc binding superoxide dismutase	COG2032P	K04565			-4.22	5.26E-09
csal0803katE	catalase	COG0753P	K03781			-2.78	4.46E-02
csal1130	alkyl hydroperoxide reductase	COG0678O				-1.99	1.96E-02
csal1861	superoxide dismutase	COG0605P	K04564	2.54	4.63E-02		
Protein repair systems							
csal0377	ribonucleoside-diphosphate reductase class Ib glutaredoxin subunit	COG0695O	K06191	1.85	1.87E-02		
csal1255	glutaredoxin-like region	COG0678O		-2.89	2.64E-03		
csal1350	protein-methionine-S-oxide reductase	COG0229O	K07305	-9.10	1.62E-04		
csal1830	glycine cleavage T protein (aminomethyl transferase)	COG0354R	K06980	-6.37	4.64E-14		
csal2129	glutaredoxin-like protein	COG0278O	K07390	2.24	1.69E-02	-11.38	1.90E-22
csal2656	Peptide methionine sulfoxide reductase MsrB (EC 1.8.4.12)	COG0229O	K07305	-23.45	3.63E-31		
csal2847	iron-sulfur cluster regulator IscR	COG1959K	K13643	2.36	4.79E-03		
csal2959	thioredoxin reductase	COG0492O	K00384	-3.41	1.11E-06		
csal3118	DSBA oxidoreductase	COG2761Q	K03673	-2.17	2.42E-03		
DNA Repair and Recombination Proteins							
csal0084	N-6 DNA methylase	COG0286V	K03427	2.23			
csal0086	restriction modification system DNA specificity protein	COG0732V		4.05			
csal0087	type III restriction enzyme, res subunit	COG4096V	K01153	3.36			
csal0224	CRISPR-associated helicase Cas3 family protein	COG1203R	K07012	1.82	2.58E-02		
csal0465	deoxyribodipyrimidine photo-lyase type I	COG0415L	K01669	-2.93	2.54E-04		
csal0718	exodeoxyribonuclease VII large subunit	COG1570L	K03601	-1.96	1.24E-02		
csal0844	type III restriction enzyme, res subunit	COG1061KL	K17677	-2.10	7.52E-03	-2.86	5.98E-05
csal0896	deoxyribodipyrimidine photolyase-like protein	COG3046R	K06876	-2.99	1.59E-05		
csal1151	endonuclease V	COG1515L	K05982	-3.37	3.62E-06		
csal1275	DNA mismatch repair protein	COG0323L	K03572	-1.77	3.26E-02		
csal1460	recombination protein RecR	COG0353L	K06187	-2.42	5.31E-04		
csal1500	radical SAM family protein	COG1533L		-2.54	9.79E-04		
csal1560	DNA topoisomerase I	COG0550L	K03168	1.97	1.72E-02	1.79	4.87E-02
csal1609	TatD-related deoxyribonuclease	COG0084L	K03424			2.43	1.45E-03
csal1621	exodeoxyribonuclease III	COG0708L	K01142	-1.83	2.59E-02		
csal1633	DNA repair protein RecO	COG1381L	K03584	-2.95	1.42E-04		
ccsal1833	excinuclease ABC subunit B	COG0556L	K03702	-3.09	5.89E-06		
cal1846	crossover junction endodeoxyribonuclease RuvC	COG0817L	K01159			2.09	7.38E-03
csal1847	holliday junction DNA helicase subunit RuvA	COG0632L	K03550			2.20	
csal2070	SOS-response transcriptional repressor, LexA	COG1974KT	K01356				
csal2865	DNA processing protein DprA	COG0758LU	K04096	-2.17	4.55E-03		
csal2972	DNA repair protein RadC	COG2003L	K03630	2.69	2.62E-03	-2.53	3.40E-04
csal3249	histone-like DNA-binding protein	COG0776L	K05787	1.84	2.00E-02		

csal3271	ATP-dependent DNA helicase UvrD	COG0210L	K03657	1.79	2.91E-02		
Redox balance							
csal0130	HcaC dioxygenase ferredoxin subunit	COG2146PR	K05710	-3.84	6.13E-07		
csal0135	glyoxalase/bleomycin resistance protein/dioxygenase	COG2514R		-10.75	1.30E-10		
csal0136	FAD-dependent pyridine nucleotide-disulfide oxidoreductase	COG0446R	K00529	-7.26	1.97E-05		
csal2376	glyoxalase/bleomycin resistance protein/dioxygenase	COG4070O		2.28	1.93E-02	-4.18	4.96E-08
csal3007	lactoylglutathione lyase	COG0346E		1.78	3.88E-02		

Table S8. Differentially expressed genes related to protein-folding stress response

Annotation nº and gene name	NCBI ANNOTATION	COG NUMBER	KO ORTHOLOGY NUMBER	fold-change 2.5 M NaCl 37ºC/ 0.6 M NaCl 37ºC	*p*-value	fold-change 2.5 M NaCl 45ºC/ 2.5 M NaCl 37ºC	*p*-value
Heat shock proteins (chaperones)							
csal0100	heat shock protein DnaJ-like protein	COG1076O	K05801			1.90	3.09E-02
csal1428	heat shock protein DnaJ-like protein	COG0484O	K05516	-3.79	1.89E-05	-2.24	2.27E-02
csal1429	heat shock protein Hsp20	COG0071O	K13993	-9.13	3.00E-06		
csal2159	chaperonin Cpn10	COG0234O	K04078	-2.61	1.24E-04		
csal2497	heat shock protein 90	COG0326O	K04079	-3.65	1.30E-07	2.13	4.22E-03
csal3093	chaperone DnaJ	COG0484O	K03686	-3.51	3.48E-05		
csal3094	chaperone DnaK	COG0443O	K04043	-16.17	7.13E-07		
csal3255	heat shock protein Hsp20	COG0071O		-4.91	6.60E-11		
csal3256	heat shock protein Hsp20	COG0071O		-1.97	9.29E-03		
Protein Degradation							
csal0499	ATPase AAA-2	COG0542O		-17.01	1.35E-06		
csal0599	ATP-dependent protease ATP-binding subunit HslU	COG1220O	K03667	-1.79	2.95E-02		
csal1279	HflK protein	COG0330O	K04088	-1.87	1.71E-02		
csal1347	heat shock protein HtpX	COG0501O	K03799	-1.79	2.69E-02		
csal2044	Lon-A peptidase	COG0466O	K01338	-3.26	1.92E-06		
csal2232	microcin-processing peptidase 1	COG0312R	K03592	-1.84	2.15E-02		
csal2234	microcin-processing peptidase 2	COG0312R	K03568	-2.65	1.21E-04		
csal2440	ATP-dependent Clp protease ATP-binding subunit ClpA	COG0542O	K03694	-4.49	8.57E-10	-1.89	2.33E-02
csal2665	PIM1 peptidase	COG0466O	K01338	-5.65	3.52E-06		
Transcriptional factors							
csa2986	RNA polymerase factor sigma-32	COG0568K	K03089	-3.77			

Table S9. Differentially expressed genes related to respiratory chain and electrochemical gradients

Annotation nº and gene name	NCBI ANNOTATION	COG NUMBER	KO ORTHOLOGY NUMBER	fold-change 2.5 M NaCl 37ºC/ 0.6 M NaCl 37ºC	*p*-value	fold-change 2.5 M NaCl 45ºC/ 2.5 M NaCl 37ºC	*p*-value
Oxidative phosphorylation							
csal0590	polyphosphate kinase	COG0855P	K00937	-2.79	6.35E-05	-1.82	4.29E-02
csal1148	flavoprotein WrbA	COG0655R	K03809			-2.98	4.85E-05
csal1313	cytochrome B561	COG3038C		-1.97	1.03E-02		
csal1412	cytochrome B561	COG3038C	K12262	-1.81	3.74E-02		
csal1569	Na^+-translocating NADH-quinone reductase subunit A	COG1726C	K00346			2.13	5.03E-03
csal1570	Na^+-translocating NADH-quinone reductase subunit B	COG1805C	K00347			1.88	2.40E-02
csal1612	electron-transferring-flavoprotein dehydrogenase	COG0644C	K00311	-2.37	8.48E-04		
csal1614	electron transfer flavoprotein subunit alpha	COG2025C	K03522	-2.04	6.17E-03	2.28	1.89E-03
csal2000	cytochrome d ubiquinol oxidase, subunit II	COG1294C	K00426	-2.38	6.69E-04		
csal2001	cytochrome bd ubiquinol oxidase, subunit I	COG1271C	K00425	-2.52	2.96E-02		
csal2281	inorganic diphosphatase	COG0221C	K01507			1.93	1.90E-02
csal2390	inorganic diphosphatase	COG0221C	K01507	-3.59	1.84E-02		
csal2801	NAD(P)H dehydrogenase (quinone)	COG2249R	K03923	-2.79	1.04E-02		
csal3121	proton-translocating NADH-quinone oxidoreductase subunit M	COG1008C	K00342	2.05	7.37E-03		
csal3122	proton-translocating NADH-quinone oxidoreductase subunit L	COG1009CP	K00341	2.36	1.86E-03		
csal3123	NADH dehydrogenase subunit K	COG0713C	K00340	1.85	3.22E-02		
csal3126	respiratory-chain NADH dehydrogenase subunit 1	COG1005C	K00337	2.33	1.21E-03		
csal3127	NADH-quinone oxidoreductase subunit G	COG1034C	K00336	2.05	7.26E-03		
csal3128	NADH-quinone oxidoreductase subunit F	COG1894C	K00335	2.10	5.04E-03		
csal3132	NADH-ubiquinone/plastoquinone oxidoreductase subunit 3	COG0838C	K00330	1.70	4.68E-02	-2.34	1.42E-03
csal3283	ATP synthase F1 subunit epsilon	COG0355C	K02114	1.88	1.49E-02	1.80	3.98E-02
csal3284	F0F1 ATP synthase subunit beta	COG0055C	K02112	2.68	7.48E-05		
csal3285	ATP synthase F1 subunit gamma	COG0224C	K02115	3.30	1.69E-06	2.36	1.30E-03
csal3286	ATP synthase F1 subunit alpha	COG0056C	K02111	2.08	4.09E-03		
csal3287	ATP synthase F1 subunit delta	COG0712C	K02113	2.44	4.43E-04		
csal3289	ATP synthase F0 subunit C	COG0636C	K02110	2.65	1.00E-04		
Porphyrin and hemo group metabolism							
csal1211	glutamyl-tRNA synthetase	COG0008J	K01885	-2.32	9.35E-04	1.97	1.38E-02
csal1326	globin	COG2346R	K06886			2.10	2.12E-02
csal2494	isochorismatase hydrolase	COG1335Q		-2.87	6.44E-05		
csal2847	BadM/Rrf2 family transcriptional regulator iscR	COG1959K	K13643	2.36			
csal2863	coproporphyrinogen III oxidase	COG0408H	K00228	-2.18	2.61E-03		
csal3299	ErpA essential respiratory protein A	COG0316S	K15724	2.05	6.56E-03		
csal3308	coproporphyrinogen III oxidase, anaerobic	COG0635H	K02495	2.28	1.41E-03		
Ubiquinone and other terpenoid-quinone biosynthesis							
csal0585	3-octaprenyl-4-hydroxybenzoate carboxy-lyase	COG0043H	K03182	3.08	1.46E-05		
csal0595	2-octaprenylphenol hydroxylase	COG0661R	K03688	-1.90	1.87E-02		
csal0845	pyrroloquinoline quinone biosynthesis protein PqqB	COG1235R	K06136	-2.05	5.69E-03		
csal0848	pyrroloquinoline quinone biosynthesis protein PqqE	COG0535R	K06139	-3.11	7.47E-06		
csal0912	putative ubiquinone biosynthesis protein	COG2941H	K06134	-2.40	6.69E-04		
csal1826	response regulator receiver domain-containing protein RegA	COG4567TK	K15012	-2.04	5.96E-03		
csal3252	4-hydroxybenzoate octaprenyltransferase	COG0382H	K03179			-1.86	3.72E-02

Sodium homeostasis							
csal0028	Na^+/solute symporter	COG0591ER		2.11	4.64E-02		
csal0255	SSS family solute/sodium (Na^+) symporter	COG0591ER				-2.70	3.25E-04
csal0400	Na^+:neurotransmitter symporter	COG0733R		2.16	3.31E-03		
csal0898	Na^+/H^+ antiporter subunit	COG1320P	K05564			-2.75	1.45E-04
csal0899	multiple resistance and pH regulation protein F	COG2212P	K05563			-3.05	8.29E-03
csal0900	cation antiporter	COG1863P				-3.15	6.07E-05
csal0901	putative monovalent cation/H^+ antiporter subunit D	COG0651CP	K05561			-3.04	2.70E-05
csal0902	putative monovalent cation/H^+ antiporter subunit C	COG1006P	K05560			-3.03	6.29E-04
csal0903	putative monovalent cation/H^+ antiporter subunit A	COG1009CP	K05559			-4.61	2.29E-09
csal1095	Na^+/H^+ exchanger	COG4651P				-3.51	7.53E-03
csal1184	Na^+/H^+ antiporter NhaC	COG1757C		1.95	2.51E-02		
csal1490	Na^+:neurotransmitter symporter	COG0733R		-1.71	4.96E-02		
csal2318	Na^+/H^+ exchanger	COG0025P		1.79	4.72E-02		
csal2891	bile acid:sodium symporter	COG0385R	K03453	1.97	1.13E-02		
csal3210	Na^+/H^+ antiporter NhaC	COG1757C		2.64	2.25E-03		
csal3274	Na^+:neurotransmitter symporter	COG0733R	K03308	3.37	2.72E-06		

Table S10. Differentially expressed genes related to chemotaxis and motility

Annotation nº and gene name	NCBI ANNOTATION	COG NUMBER	KO ORTHOLOGY NUMBER	fold-change 2.5 M NaCl 37ºC/ 0.6 M NaCl 37ºC	*p*-value	fold-change 2.5 M NaCl 45ºC/ 2.5 M NaCl 37ºC	*p*-value
Regulation							
csal1980	anti-sigma-28 factor FlgM	COG2747KNU	K02398			2.14	5.47E-03
csal2011	sigma 28 (flagella/sporulation)	COG1191K	K02405			14.02	4.68E-25
csal2027	transcriptional activator FlhC	-	K02402	1.97	9.36E-03	6.44	3.76E-14
csal2028	putative flagellar transcriptional activator transcription regulator protein	-	K02403	1.84	2.07E-02	1.90	2.20E-02
Motor/switch							
csal1504	OmpA/MotB protein	COG1360N	K02557	-2.63	3.26E-03	5.45	9.49E-08
csal1505	MotA/TolQ/ExbB proton channel	COG1291N	K02556			5.18	4.41E-09
csal2025	OmpA/MotB protein	COG1360N	K02557	1.93	1.52E-02	11.93	2.87E-22
csal2026	chemotaxis MotA protein	COG1291N	K02556	2.67	1.50E-04	13.09	3.32E-24
Flagellar assembly proteins							
Type-III secretion							
csal1957	flagellar assembly protein FliH	COG1317NU	K02411			33.89	1.74E-30
csal1958	ATPase FliI/YscN	COG1157NU	K02412			12.42	1.47E-19
csal1964	flagellar biosynthesis protein, FliO	COG3190N	K02418			5.01	5.18E-07
csal1965	flagellar biosynthesis protein FliP	COG1338NU	K02419			5.93	2.35E-11
csal1966	flagellar biosynthetic protein FliQ	COG1987NU	K02420			2.14	1.02E-02
csal1967	flagellar biosynthetic protein FliR	COG1684NU	K02421	-3.31	3.12E-04	5.10	7.27E-07
csal2012	hypothetical protein	-	K03516			4.66	1.16E-08
csal2014	GTP-binding signal recognition particle SRP54, G-protein	COG1419N	K02404			6.90	8.92E-13
csal2015	flagellar biosynthesis protein FlhA	COG1298NU	K02400			7.90	2.25E-14
csal2016	flagellar biosynthetic protein FlhB	COG1377NU	K02401			13.09	2.06E-22
C-ring							
csal1956	flagellar motor switch protein FliG	COG1536N	K02410	1.84	2.45E-02	26.85	2.00E-35
csal1962	flagellar motor switch protein FliM	COG1868N	K02416			6.54	2.19E-12
csal1963	flagellar motor switch FliN	COG1886NU	K02417			6.15	5.00E-11
M,S,P and L rings							
csal1955	flagellar M-ring protein FliF	COG1766NU	K02409			14.27	9.32E-19
csal1971	flagellar basal body P-ring protein	COG1706N	K02394			16.60	1.27E-27
csal1972	flagellar L-ring protein	COG2063N	K02393			22.51	1.39E-30
csal1979	flageller protein FlgA	COG1261NO	K02386			26.91	2.58E-35
Rod,hook and filament							
csal0506	flagellar hook-associated 2-like protein	COG1345N		4.52	2.32E-09		
csal1954	flagellar hook-basal body complex protein (FliE)	COG1677NU	K02408	-2.11	5.57E-03	2.99	3.92E-05
csal1960	flagellar hook-length control protein	COG3144N	K02414	2.17	4.04E-03	8.70	2.15E-17
csal1961	flagellar basal body-associated protein FliL	COG1580N	K02415	3.12	1.68E-05	10.44	4.68E-20
csal1968	flagellar hook-associated protein FlgL	COG1344N	K02397			5.10	4.63E-11
csal1969	flagellar hook-associated protein	COG1256N	K02396			8.51	5.77E-18
csal1970	mannosyl-glycoprotein endo-beta-N-acetylglucosamidase	COG1705NU	K02395			16.19	1.77E-23
csal1973	flagellar basal-body rod FlgG	COG4786N	K02392			21.86	7.01E-32
csal1974	flagellar basal-body rod FlgF	COG4787N	K02391			26.47	2.54E-05
csal1975	flagellar basal body FlaE	COG1749N	K02390	1.93	2.64E-02	22.57	3.05E-27
csal1976	flagellar basal body rod modification protein	COG1843N	K02389	1.99	9.85E-03	27.84	1.42E-36
csal1977	flagellar basal-body rod protein FlgC	COG1558N	K02388			13.16	4.21E-21

csal1978	flagellar basal-body rod protein FlgB	COG1815N	K02387			12.59	2.75E-14
csal1985	flagellin-like protein	COG1344N	K02406	5.13	4.05E-11	10.39	1.99E-21
csal1993	flagellar protein FlaG protein	COG1334N	K06603			2.42	1.39E-03
csal2033	flagellar hook-associated 2-like protein	COG1345N	K02407			5.74	2.24E-12
Chaperones							
csal1959	flagellar export FliJ	COG2882NUO	K02413	2.55	1.14E-03	16.25	4.68E-25
csal1981	FlgN protein	COG3418NUO	K02399			2.59	4.41E-04
csal2031	hypothetical protein	-	K02423	-6.40	1.29E-13	4.04	2.61E-08
csal2032	flagellar protein FliS	COG1516NUO	K02422			4.72	1.67E-08

Table S11. Differentially expressed genes related to iron homeostasis

Annotation nº and gene name	NCBI ANNOTATION	COG NUMBER	KO ORTHOLOGY NUMBER	fold-change 2.5 M NaCl 37ºC/ 0.6 M NaCl 37ºC	*p*-value	fold-change 2.5 M NaCl 45ºC/ 2.5 M NaCl 37ºC	*p*-value
Iron transport and metabolism							
csal0549	ferric iron ABC transporter, iron-binding protein	COG1840P	K02012	2.72	6.28E-05		
csal1040	FIG00546271: hypothetical protein	COG4759O		2.98	4.68E-06		
csal1041	ABC-type Fe3+-siderophore transport system, permease 2 component	COG0609P	K02015	6.48	1.99E-04		
csal1042	iron(III) dicitrate transport system permease protein FecD (TC 3.A.1.14.1)	COG0609P	K02015	4.71	8.39E-08		
csal1043	periplasmic binding protein	COG0614P	K02016	12.36	1.09E-23		
csal1044	TonB-dependent receptor	COG1629P	K02014	14.14	8.35E-26		
csal1045	ferric hydroxamate outer membrane receptor FhuA	COG4773P	K02014	1.70	4.81E-02		
csal1056	aerobactin siderophore receptor IutA	COG1629P	K02014	7.46	6.44E-05		
csal1502	TonB-dependent receptor	COG4206H	K16092	2.46	4.99E-02		
csal2539	TonB-dependent siderophore receptor	COG4773P	K16088	2.80	7.12E-05	-2.30	2.50E-03
csal2549	ABC transporter (iron.B12.siderophore.hemin) , ATP-binding component	COG1120PH		-3.15	3.85E-06	-1.78	4.17E-02
csal2678	TonB-dependent receptor; Enterobactin receptor IrgA	COG4771P	K16089	-1.79	2.70E-02		
csal2699	iron(III) dicitrate transport system, periplasmic iron-binding protein FecB (TC 3.A.1.14.1)	COG0614P	K02016			2.04	8.43E-03
csal2700	ABC-type Fe3+-siderophore transport system, permease component	COG0609P	K02015			2.71	2.40E-04
csal2701	ABC-type Fe3+-siderophore transport system, permease 2 component	COG4779P	K02015			3.35	2.07E-05
csal2702	ABC-type Fe3+-siderophore transport system, ATPase component	COG1120PH	K02013	-2.26	1.98E-02	2.92	2.51E-03
csal3089	periplasmic binding protein	COG0614P	K02016	-1.87	1.67E-02	-2.05	8.04E-03
csal3091	ABC transporter related	COG1120PH	K02013	-2.29	1.37E-03		
csal3182	putative OMR family iron-siderophore receptor precursor	COG4773P	K16088	5.29	1.22E-11		
csal3258	ferrichrome-iron receptor	COG4774P	K02014	21.81	7.74E-33	-53.25	3.66E-03
csal3311	ferrous iron transport periplasmic protein EfeO	COG2822P	K07224			-3.76	3.17E-07
csal3312	ferrous iron transport peroxidase EfeB	COG2837P	K16301			-2.53	3.40E-04
Siderophore biosynthesis							
csal1053	siderophore biosynthesis complex, short chain	COG4264Q		21.81	7.74E-33	-31.65	4.96E-38
csal1054	siderophore biosynthesis protein, monooxygenase	COG3486Q	K03897	18.17	7.74E-33	-28.14	3.91E-22
csal1055	siderophore biosynthesis L-2,4-diaminobutyrate decarboxylase	COG0076E	K13745	14.88	9.65E-29	-28.65	1.02E-36
csal1595	periplasmic serine proteases (ClpP class)	COG02650	K01362	-6.44	4.18E-14		
csal1628	serine protease precursor MucD/AlgY	COG0616OU	K04773	-2.84	6.28E-05		
csal1779	isochorismatase hydrolase	COG1335Q		-4.58	1.08E-09	-2.71	1.84E-04
csal2494	isochorismatase hydrolase	COG1335Q		-2.87	6.44E-05		
Ferrous iron binding							
csal2129	glutaredoxin-like protein	COG0278O	K07390	2.24	1.69E-02	-11.38	1.90E-22
csal3257	ferric iron reductase	COG4114R	K13255	7.06	2.37E-15		
csal0133	short chain dehydrogenase	COG1028IQR		-3.30	2.10E-04		
csal2900	bacterioferritine	COG2906P	K02192	-1.94	1.05E-02		
csal2953	ferritin Dps	COG0783P	K04047	-5.19	1.36E-11		
Iron homeostasis regulation							
csal1052	sigma-24 (FecI-like protein)	COG1595K	K03088	11.96	5.13E-10		
csal1098	sigma-24 (FecI-like protein)	COG1595K	K03088	-1.78	3.82E-02		
csal1449	sensor protein	COG1840P	K02012	-3.30	2.13E-04		
